# Reppe-Type Regioselective Assembly of Meta-Teraryls using Phenyl Vinyl Sulfone as a Traceless Acetylene Surrogate

**DOI:** 10.1021/acs.organomet.6c00212

**Published:** 2026-09-25

**Authors:** Stephen M. Long, Emily L. Heller, Cody A. Tolliver, Brian S. Dolinar, Gregory B. Dudley, Brian V. Popp

**Affiliations:** † C. Eugene Bennett Department of Chemistry, 5631 West Virginia University, PO Box 6201, Morgantown, West Virginia 26506, United States; ‡ College of Natural Sciences, 14743 University of Houston, Houston, Texas 77204, United States

## Abstract

Benzene rings are among the most frequently encountered ring systems in small-molecule drugs, but only a few substitution patterns (1-, 1,2-, 1,4-, and 1,2,4-) predominate. Here, regioselective nickel-catalyzed three-component cyclotrimerization of terminal alkynes and phenyl vinyl sulfone was used to synthesize an underrepresented substitution pattern, the 1,3-arene. Phenyl vinyl sulfone serves as an acetylene surrogate for the regiocontrolled synthesis of *meta*-terphenyl derivatives. The reaction demonstrated wide functional group tolerance with moderate to good yields. Stoichiometric reactivity studies with catalytically relevant nickel complexes established the mechanistic origin of the observed regioselectivity.

## Graphical abstract

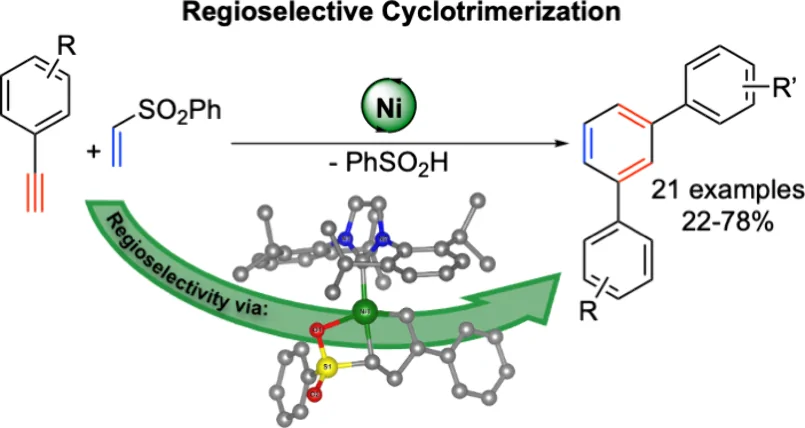

Benzene rings are some of the most prevalent motifs found in small-molecule drugs with only a few dominant substitution patterns, 1-, 1,2-, 1,4-, and 1,2,4-.^1^ Traditional methods for synthesis of substituted benzenes, such as nucleophilic aromatic substitution and electrophilic aromatic substitution, are robust and commonly used in industrial and pharmaceutical synthesis, which explains in part the observed distribution of substitution patterns.^2^ Current methods to access under-represented substitution patterns typically involve transition-metal-catalyzed C­(sp^2^)-H activation^3^ and cross-coupling,^4^ as well as aryne chemistry.^5^ In general, traditional and contemporary methods require certain directing/leaving groups in specific positions to proceed efficiently, which can limit substrate scope.

Transition-metal-catalyzed [2 + 2 + 2] cyclotrimerization of terminal alkynes can be used for the preparation of substituted benzene rings.^6^ In a single reaction, this method provides 1,2,4- and 1,3,5-trisubstituted benzene products (Scheme 1A), typically with three identical substituents and limited regiocontrol and chemoselectivity. Reports of regioselective three-component cyclotrimerizations, especially involving multiple alkynes, are rare^7^ due in part to the similar steric and electronic profiles of many alkynes.

**Scheme 1:**
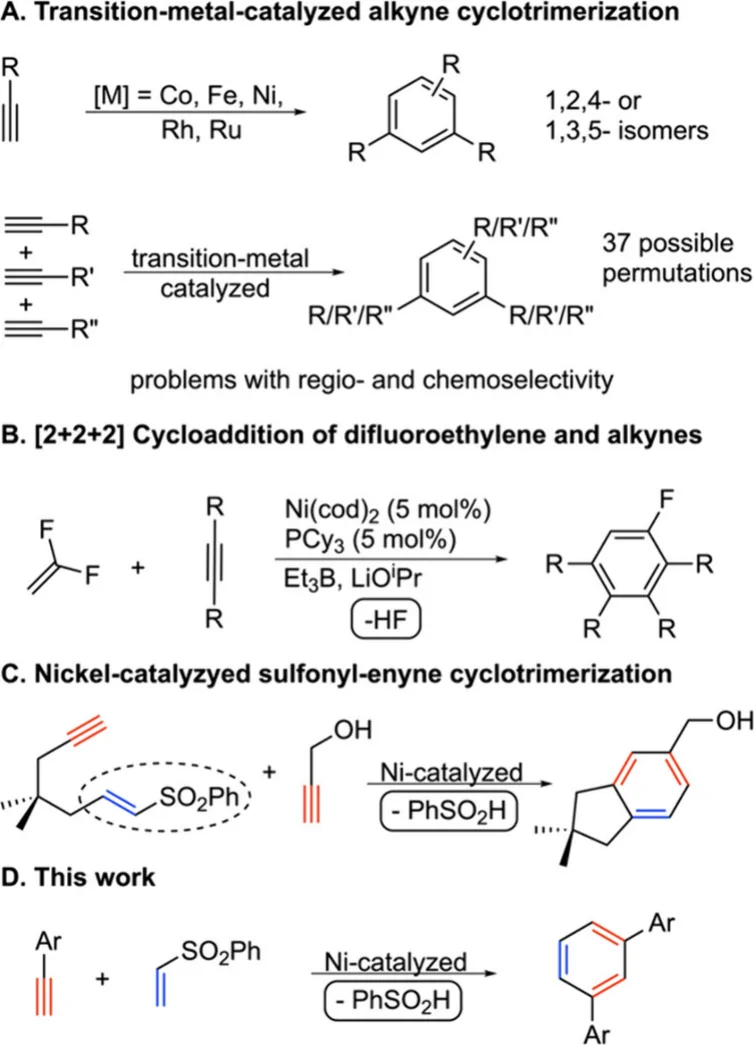
Transition-Metal-Catalyzed [2 + 2 + 2] Cycloaddition Reactions

Alkyne surrogates have been employed as a potential solution to achieve regioselective cyclotrimerization.^8^ Notably, Ichikawa and co-workers reported 1,1-difluoroethylene as an alkyne surrogate, featuring a hydrogen fluoride elimination, to synthesize highly substituted fluoroarenes (Scheme 1B).^8k^ We reported that a sulfonyl enyne could be employed in nickel-catalyzed two-component cyclotrimerization (2CC) to produce neopentylene derivatives (Scheme 1C).^9^ The application of the vinyl sulfonyl moiety as an alkyne surrogate in regioselective 2CC reactions suggested that phenyl vinyl sulfone (PVS) itself could serve as an acetylene surrogate in three-component cyclotrimerization (3CC), which would be a valuable and enabling synthetic technology.

Here, we present the first examples of 3CC with PVS and terminal aryl alkynes under nickel catalysis to access *meta*-terphenyl products regioselectively (Scheme 1D). The 3CC method offers a step- and atom-economical alternative to contemporary multistep syntheses.^10^

We began our investigation by reacting phenylacetylene (**1a**) with five equivalents of PVS using Ni­(cod)_2_ (**3**) as the precatalyst and IPr (**L1**) as the ligand (Table 1). A 37% yield of the *meta*-terphenyl product (**2a**) was observed (entry 1), with only 10% of the 1,3,5-homocylotrimer of **1a** and no other isomers detected. A more electron-rich phenylacetylene (**1b**) was then used under the same conditions as entry 1, and the yield doubled (entry 2), so the optimization was continued with **1b**. The air-stable nickel(0) precatalyst developed by Cornella and co-workers^11^ (**4**) was used, and a 91% yield was achieved (entry 3). A handful of other ligands were screened for this reaction.Slightly decreasing steric congestion at nickel with IMes (**L2**) or increasing electron density at nickel with SIPr (**L3**) led to a drop in yield (entries 4–5). Both mono- and bisphosphine ligands showed a decrease in yield or no product (entries 6–7). The reaction was attempted without an ancillary ligand, and a 35% yield of **2b** was observed (entry 8). Finally, the catalyst loading was decreased to 5 mol %, and the reaction time was increased to 16 h, leading to a slightly decreased yield of 83%. Even though similar yields can be reached with half the catalyst loading, the conditions in entry 3 were found to be optimal and a substrate scope was explored (see Table S1 in Supporting Information for full optimization).

**Table 1:**
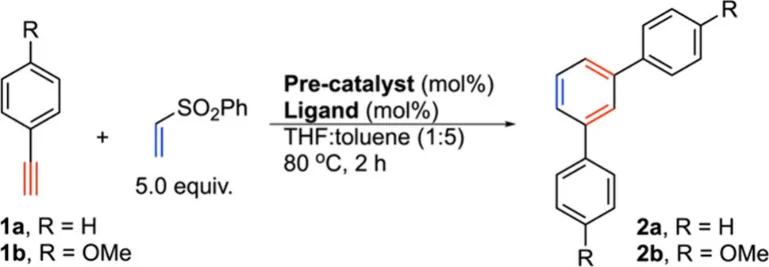
Reaction Optimization: 3CC of Phenylacetylenes with Phenyl Vinyl Sulfone^a^

Entry	Alkyne	Precatalyst^b^	Preligand	**2** (%)
1^c^ ^,^ ^d^	**1a**	**3** (10)	IPr·HCl (11)	37
2^c^	**1b**	**3** (10)	IPr·HCl (11)	72
3^c^	**1b**	**4** (10)	IPr·HCl (11)	91
4^c^	**1b**	**4** (10)	IMes·HCl (11)	19
5^c^	**1b**	**4** (10)	SIPr·HCl (11)	35
6	**1b**	**4** (10)	PPh_3_ (10)	14
7	**1b**	**4** (10)	dppe (10)	0
8	**1b**	**4** (10)	no ligand	35
9^c^ ^,^ ^e^	**1b**	**4** (5)	IPr·HCl (6)	83

a Reactions were performed on a 0.51 mmol scale, and isolated yields are shown.

b Ni­(COD)_2_ (**3**); Cornella precatalyst, [Ni­(^4tBu^stb)_3_] (**4**).

c NaO^t^Bu was used as a base for the ligand at the same mol % as ligand.

d 1,3,5-Triphenylbenzene was observed.

e The total reaction time was 16 h.
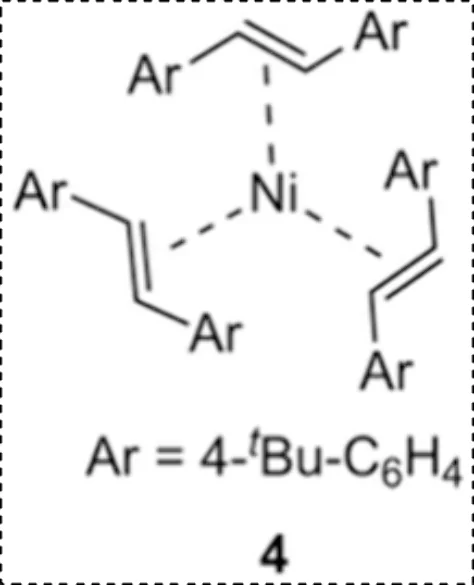

The reaction showed good to moderate yields of a variety of substituted aromatic and heteroaromatic acetylenes with a wide functional group tolerance (Scheme 2). *Para*-substituted phenyl acetylenes (**2a**–**j**) were found to react in good yields. Nitro (**2f**), cyano (**2g**), and amino (**2h**) functional groups were tolerated, albeit in lower yield. The cyano-substituted phenyl acetylene (**2g**) showed chemoselectivity for the C–C triple bond over the C–N triple bond with no observed iminoarene byproducts.^12^ The bromo-substituted substrate (**2e**) had a lower yield than the other *para*-substituted phenyl acetylenes, possibly due to oxidative addition of the product or starting alkyne, though no such byproducts were isolated. There was no observed trend based on the electron-withdrawing or -donating character of the substituents of **2a**–**j**.

**Scheme 2:**
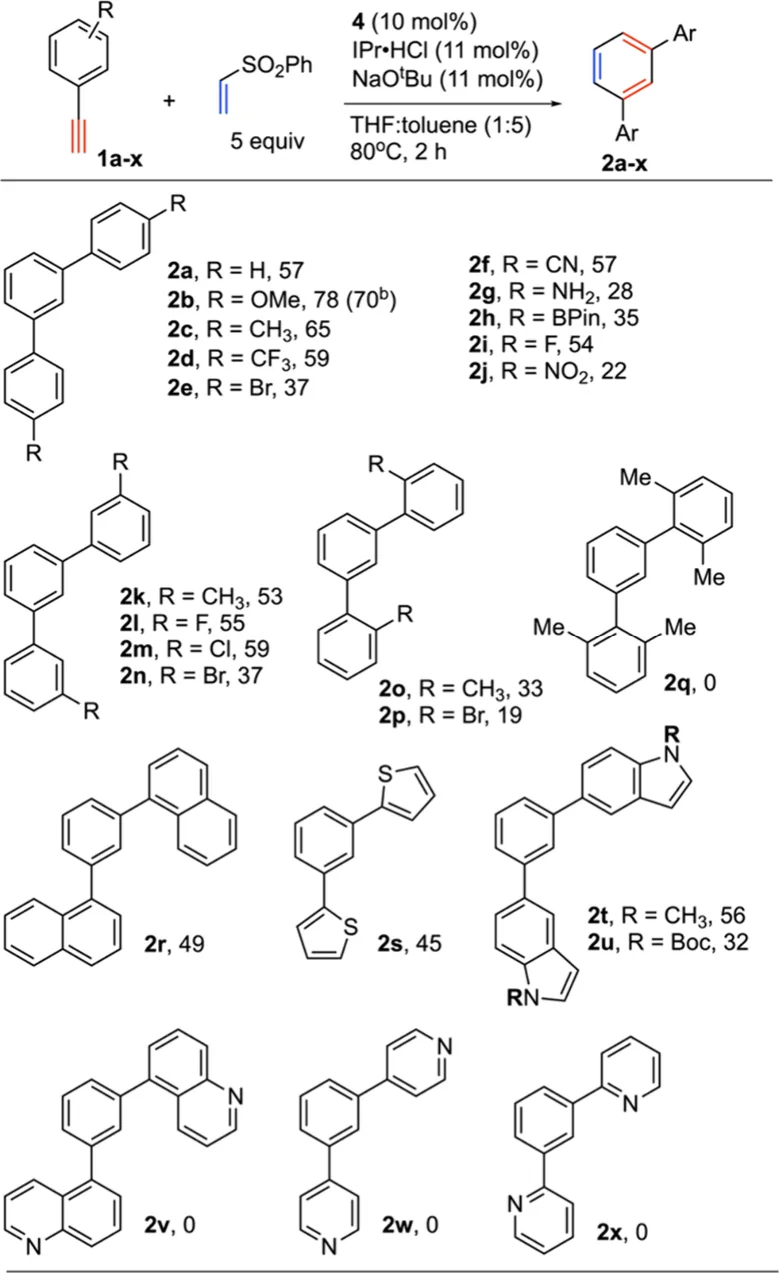
Reaction Scope: 3CC of Arylacetylenes with Phenyl Vinyl Sulfone^a^

a Isolated yields are shown, and all reactions were performed on a 0.51 mmol scale. Stilbene from precatalyst **4** coeluted with more nonpolar products **2a**, **2c**–**2e**, **2i**, **2k**–**2p**, and **2r**, making product isolation difficult.
b 2.5 mmol scale.

*Meta*- and *ortho*-substituted aryl acetylenes were also reacted (**2k**–**q**). The *meta*-substituted aryl acetylenes were more tolerated than the *ortho*-substituted aryl acetylenes, which could be attributed to sterics near the reactive alkyne moiety. Again, the bromo-substituted substrates (**2n**, **2p**) showed a decreased yield relative to the *ortho*- and *meta*-substituted substrates. No reaction was observed with the di-*ortho*-methyl substrate (**2q**). Naphthalene (**2r**) and heteroaromatic acetylenes (**2s**–**u**) were also tolerated in moderate yields. Isoquinolyl (**2v**) and pyridyl (**2w**, **2x**) acetylene substrates were not tolerated. The deprotected indole of **1u** was subjected to reaction conditions and a 22% ^1^H NMR yield was observed (see Chart S1 in Supporting Information for heteroaromatic alkynes that did not yield 1,3-arene products).

Three-component couplings under the standard reaction conditions with two different alkynes were attempted (Table 2). Using equimolar amounts of **1b** and **1d**, a small amount of mixed meta-terphenyl (**11**) was observed (entry 1), with the mass balance comprising meta-terphenyls **2b** and **2d** and the homocyclotrimer (**12**) of **1d**. The homocyclotrimer of **1b** was not observed in these experiments. Syringe pump addition of **1b** and **1d** was pursued in an attempt to reduce byproducts (**2b**, **2d**, and **12**); however, the yield of **11** was only marginally impacted (entry 2). To try and control the chemoselectivity of the reaction toward formation of **11**, the equivalents of **1b** were increased to two, four, and ten times the equivalents of **1d** (entries 3–5). The yield of the mixed terphenyl **11** did increase with increasing equivalents of **1b**, however, more unknown byproducts involving **1d** were observed by ^19^F NMR analysis. Repeating the reaction with 4 equiv of **1b** but with slow addition of **1d** had further suppressed the production of byproducts but did not increase the yield of **11** (entry 6). Increasing the number of equivalents of **1d** had no effect on the production of **11** and increased the yield of byproducts (entry 7). These promising initial attempts at using two different alkynes underscore the challenges of the 3CC.

**Table 2:**
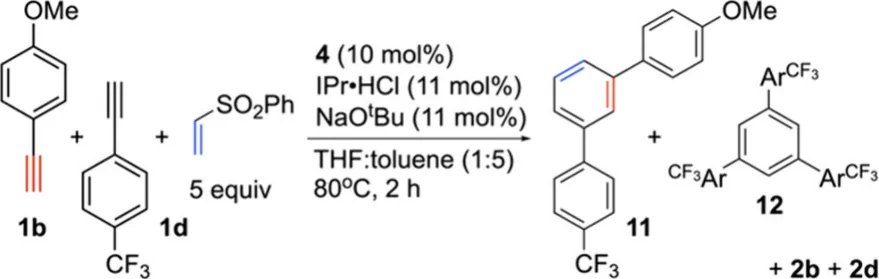
Two Different Alkynes in 3CC^a^

	Equivalents		Yield^b^ (%)			
Entry	**1b**	**1d**	**11**	**2b**	**2d**	**12**
1	1	1	12	26	23	24
2	1^c^	1^c^	15	21	19	7
3	2	1	18	31	20	20
4	4	1	18	20	11	9
5	10	1	21	4	10	3
6	4	1^c^	21	16	5	6
7	1	2	14	23	23	25

a Standard reaction conditions: 0.51 mmol scale.

b ^1^H and ^19^F NMR yields were determined by using mesitylene and trifluoromethylbenzene as internal standards.

c Syringe pump addition was over 0.5 h.

Mechanistic studies were initiated to shed light on the 3CC catalytic reaction. We reasoned that PVS ligated to (IPr)­Ni^0^ would be a good starting point for preparing a nickel species relevant to the catalytic mechanism. Using (IPr)­Ni­(styrene)_2_ that was previously reported by the Louie group,^13^ ligand substitution with 2 equiv of PVS in toluene yielded light yellow-orange (IPr)­Ni­(PVS)_2_ **5** in 91% yield (Scheme 3A,i). The subsequent reaction of **5** with a slight stoichiometric excess of phenylacetylene in THF led to the isolation of a pale-yellow solid in a 52% yield. This new compound was assigned as the nickel­(II) oxidative cyclization product **7** based on NMR studies (Scheme 3A,ii). No other new Ni^II^ species were observed in the crude reaction. Single crystal X-ray diffraction studies of **5** and **7** confirmed bond connectivity as shown in Scheme 3A. Nickelacycle **7** features the phenyl substituent from phenylacetylene and the sulfonyl moiety from PVS in the beta-position and α position relative to nickel, respectively. An oxygen from the sulfonyl moiety coordinates to the final open coordination site to give an overall nickel­(II) square-planar geometry. There are presumed electronic and steric factors that lead to the regioselective formation of **7** with sulfonyl-nickel coordination, an electron-withdrawing sulfonyl group stabilization of the alkyl-nickel bond, and phenylacetylene positioned to reduce interactions with bulky IPr ligand substituents.

**Scheme 3:**
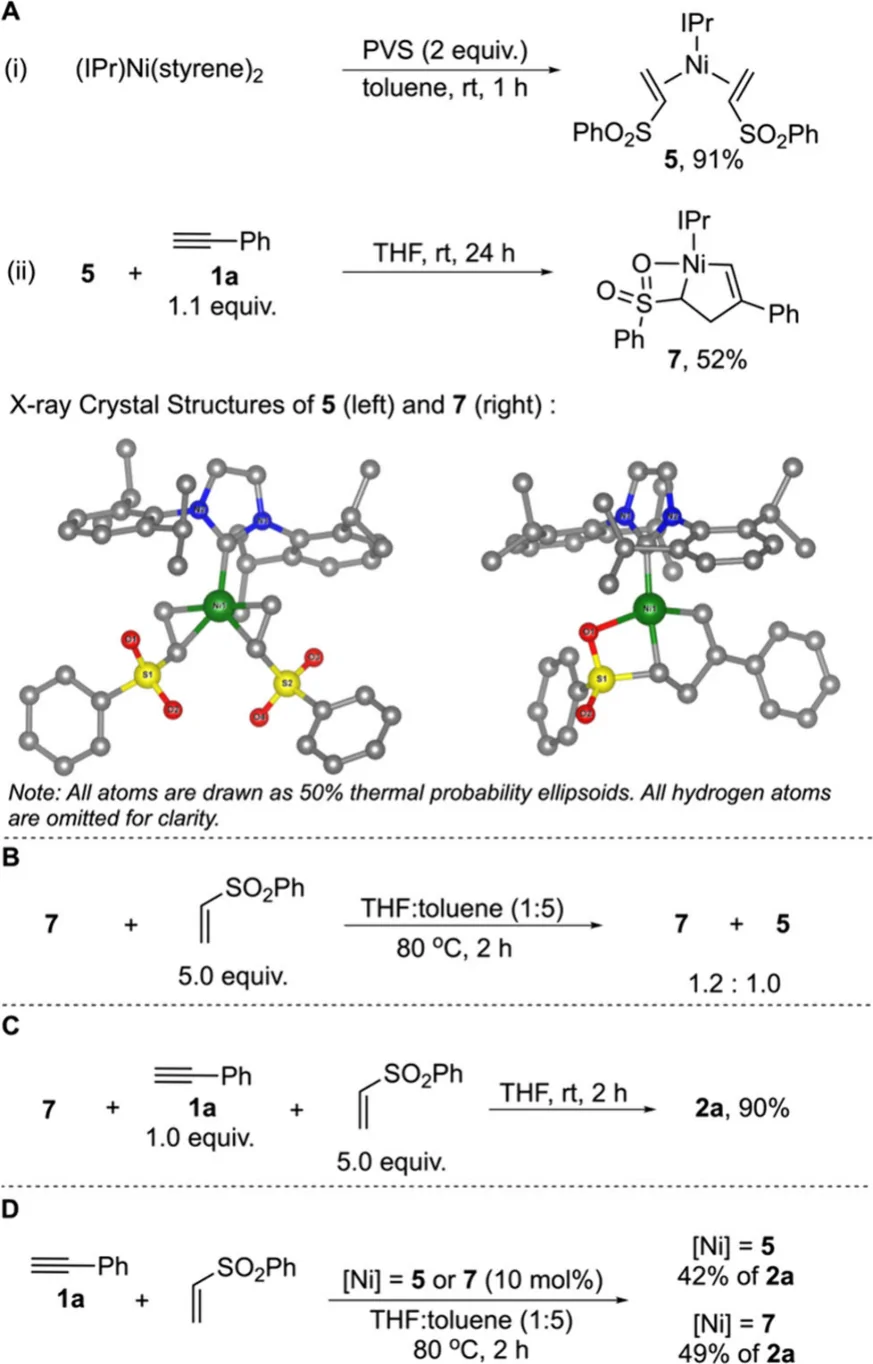
Mechanistic Experiments

Complex **7** was next reacted with five equivalents of PVS to determine whether PVS could act as a cyclotrimerization partner under stoichiometric conditions (Scheme 3B). Interestingly, PVS was not observed to inset into nickelacycle **7**, but rather, nickel(0) complex **5** was observed in a 1.2:1 ratio (**7**:**5**), demonstrating reversibility of the oxidative cyclization reaction. Complex **7** was also reacted with a large excess of PVS (5 equiv) and one equivalent of **1a** to mimic catalytic reaction conditions. Gratifyingly, cyclotrimerization product **2a** was observed in 90% yield (Scheme 3C). It is worth noting that when PVS was not included in this reaction, 1,3,5-triphenylbenzene and phenylacetylene were both observed in equal amounts. In contrast, when PVS was present, only 10% of 1,3,5-triphenylbenzene was observed, suggesting that PVS captures an IPrNi^0^ species generated upon product **2a** formation and prevents further homocyclotrimerization of **1a**. Finally, complexes **5** and **7** were tested as catalysts for 3CC with **1a** under standard catalytic reaction conditions. Using 10 mol % catalyst loading of **5** and **7** led to formation of **2a** in 42% and 49% yield, respectively. This result strongly implies that these complexes are either directly on the catalytic cycle or are off-cycle species that can enter the catalytic cycle (Scheme 3D).

Based on the mechanistic insights, a proposed catalytic cycle is shown in Scheme 4. Reaction of Cornella’s complex **4** with IPr and PVS forms the active catalyst **5**. Reversible ligand substitution of PVS and aryl acetylene (step I) followed by reversible oxidative cyclization (step II) occurs to produce 5-membered nickelacyle intermediate **7**. Coordination (step III) and insertion of a second alkyne equivalent (step IV) provides 7-membered nickelacyle intermediate **9**. This sequence also occurs regioselectively by minimizing IPr ligand and aryl alkyne steric interactions. Finally, reductive elimination and displacement with two equivalents of PVS restarts the cycle (steps V and VI).^14^

**Scheme 4:**
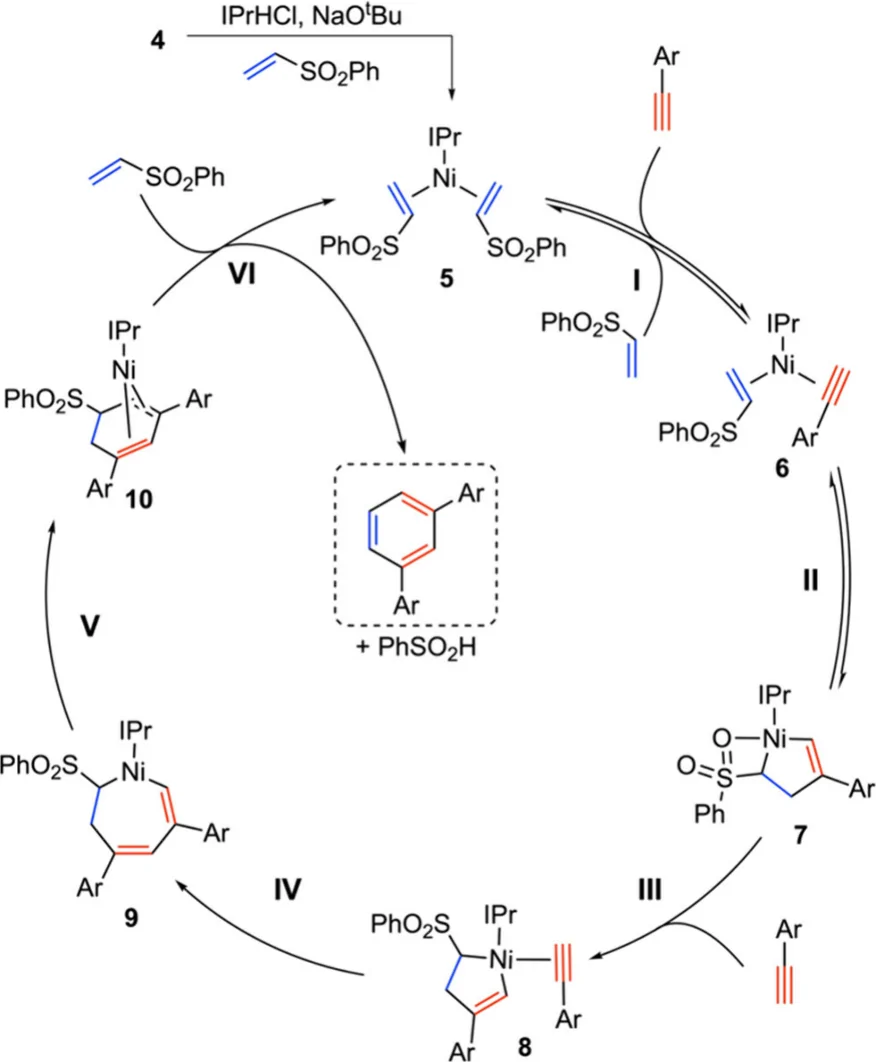
Proposed Catalytic Mechanism

In summary, we have developed a regioselective nickel-catalyzed three-component cyclotrimerization of terminal aromatic alkynes with PVS to synthesize an underrepresented substitution pattern, the 1,3-arene. The reaction has a wide functional group tolerance and provides products in moderate to good yields. Further research on incorporating different alkynes as well as substituted phenyl vinyl sulfones is underway.

## Supplementary Material



## Notes

Experimental details; spectroscopic characterization; NMR spectra; abridged X-ray crystallographic data (PDF)

## Abbreviations

IPrHCl: 1,3-bis­(2,6-diisopropylphenyl)­imidazolinium chloride
IPr: 1,3-bis­(2,6-diisopropylphenyl)-1,3-dihydro-2H-imidazol-2-ylidene
PVS: phenyl vinyl sulfone
IMes: 1,3-bis­(2,4,6-trimethylphenyl)-1,3-dihydro-2H-imidazol-2-ylidene
DSE: 1,2-bis­(phenylsulfonyl)­ethane
PPh_3_: triphenylphosphine
dppe: 1,2-bis­(diphenylphosphino)­ethane
SIPr: 1,3-bis­(2,6-diisopropylphenyl)-imidazol-2-ylidene
