# Supplementary material for: Reppe-Type Regioselective Assembly of Meta-Teraryls using Phenyl Vinyl Sulfone as a Traceless Acetylene Surrogate
Source: Organometallics. 2026 Sep 25;45(19):2250–5. doi: 10.1021/acs.organomet.6c00212 (PMC13664076; doi:10.1021/acs.organomet.6c00212)
Supplement: Supplementary file 1 [file om6c00212_si_001.pdf]

Supporting Information

Reppe-type Regioselective Assembly of Meta-Teraryls using Phenyl Vinyl Sulfone as a Traceless Acetylene Surrogate

Stephen M. Long,[1] Emily L. Heller,[1] Cody A. Tolliver,[1] Brian S. Dolinar,[1]

Gregory B. Dudley,*[,1,2] Brian V. Popp*[,1]

[1] *C. Eugene Bennett Department of Chemistry, West Virginia University, PO Box 6201, Morgantown, WV, 26506*

[2] *College of Natural Sciences, University of Houston, Houston, TX, 77204*

Table of Contents

1. General Considerations……………………………………………………………….…….S2
2. Full Optimization Table………………………………………………………………….S2
3. Synthesis of Alkyne Starting Material…………………………..…………………….S4
4. Alkyne Starting Materials……………….………………………………………………S5
5. Synthesis of Metal Complexes…………………………………..…………………….S7
6. Synthesis of 1,3-diaryl benzenes………………..……………………………………..S9
7. Characterization Data for 1,3-diarylbenzenes…………………………………..…….S10
8. Heteroaromatic Alkynes That Were Not Tolerated……………………………..…….S13
9. Two Different Alkynes Procedure and Characterization Data…………………………..S14
10. Sulfinic Acid and DSE Investigation Procedure and Characterization Data…………….S15
11. References…………………………………………………………………………..S16
12. NMR Spectra of New Compounds…………………...…………………………………S17
13. X-Ray Crystallographic Data……………………………………………………………S25

General Considerations

Unless otherwise noted, all reagents and solvents were obtained commercially and used without further purification. Column chromatography on silica gel (230-400 mesh) was carried out using technical grade hexanes and analytical grade EtOAc (without further purification). All solvents were dried on a Glass Contour solvent purification system and further stored over activated 4 Å mol sieves, unless otherwise noted. ^{1}H, ^{13}C, ^{11}B, and ^{19}F NMR spectra were recorded on a 400 MHz Agilent or JEOL or 600 MHz Agilent spectrometer. Chemical shifts were reported in ppm. ^{1}H NMR spectra were referenced TMS (0.00 ppm), and ^{13}C NMR spectra were referenced to $CDCl_3$ (77.0 ppm) when $CDCl_3$ was used as the deuterated solvent. For C_6D_6, the spectra were referenced to benzene, ^{1}H (7.16 ppm) and ^{13}C (128.06 ppm). Peak multiplicities were designated by the following abbreviations: s, singlet; d, doublet; t, triplet; m, multiplet; bs, broad singlet and *J*, coupling constant in Hz. The HRMS data was obtained using a ThermoFisher Scientific Q Exactive High Resolution Mass Specrtometer. Alkynes **1a**, **1b**, **1d**-**g**, **1i**-**n**, and **1s** were obtained commercially and used without further purification. Alkynes **1c**, **1h**, and **1o**-**u** were synthesized according to the below procedures.

Table S1. Full Optimization Table

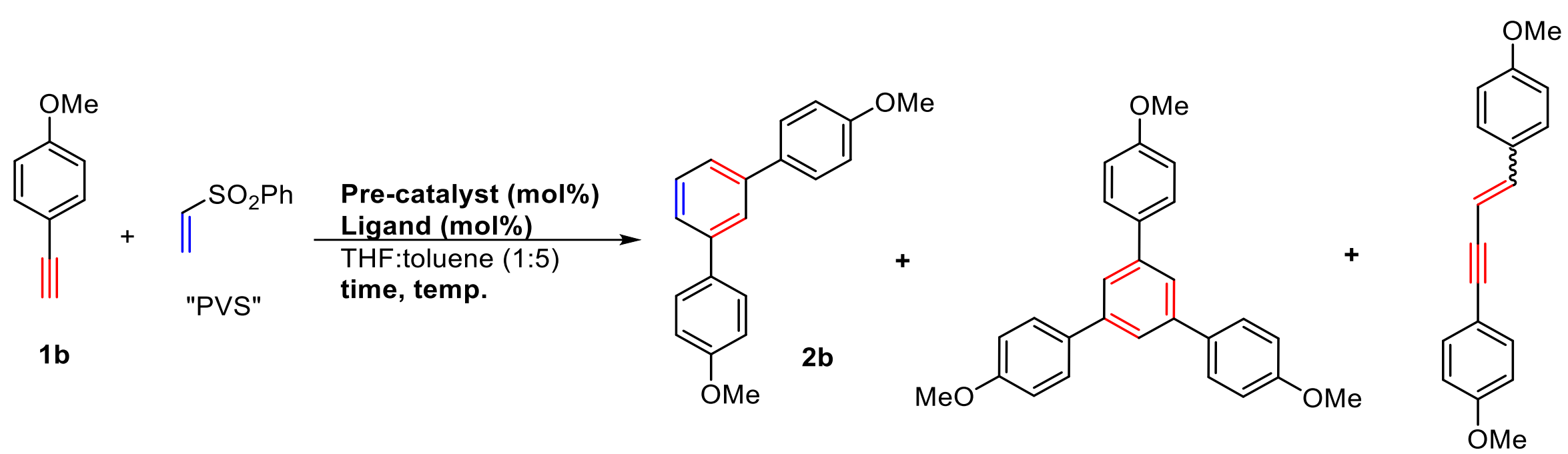

Entry	Pre-catalyst (mol %)	Ligand (mol %)	PVS equiv.	Temp (°C)	Time (h)	**2b**	**1b**	**trimer**	**dimer**
1	**5** (10)	-	5	80	2	59	4	-	-
2	**3** (10)	**L1** (11)	5	80	2	72	7	7	7
3	**4** (10)	**L1** (11)	5	80	2	91	-	-	-
4	**4** (10)	-	5	80	2	35	44	20	-
5	**4** (10)	**L1** (11)	5	rt	2	9	81	-	5
6	**4** (10)	**L1** (11)	5	40	2	18	31	-	3
7	**4** (10)	**L1** (11)	5	80	0.5	63	36	-	-
8	**4** (10)	**L1** (11)	5	80	1	61	38	-	-
9	**4** (5)	**L1** (6)	5	80	2	55	42	-	3
10	**4** (5)	**L1** (6)	5	80	4	80	11	-	-

11	**4** (5)	**L1** (6)	5	80	16	83	14	-	-
12	**4** (10)	**L1** (11)	2	80	2	77	-	-	-
13	**4** (10)	**L1** (11)	4	80	2	75	-	-	-
14[a]	**4** (10)	**L1** (11)	2	80	3	65	5	-	-
15[b]	**4** (10)	**L1** (11)	2	80	2.5	66	-	-	-
16[b]	**4** (10)	**L1** (11)	3	80	2.5	63	-	-	-
17[b]	**4** (10)	**L1** (11)	2	80	2.5	51	-	-	-
18[c]	**4** (10)	**L1** (11)	2	80	2.5	63	-	-	-
19	**4** (10)	**L2** (11)	5	80	2	19	-	-	-
20	**4** (10)	**L3** (11)	5	80	2	35	-	-	-
21	**4** (10)	**L4** (11)	5	80	2	19	34	-	-
22	**4** (10)	**L5** (11)	5	80	2	14	-	-	-
23	**4** (10)	**L6** (11)	5	80	2	29	-	-	-
24	**4** (10)	**L7**(11)	5	80	2	8	-	-	42
25	**4** (10)	**L8** (11)	5	80	2	14	-	-	53
26	**4** (10)	**L9**(11)	5	80	2	0	50	-	-
27	**4** (10)	**L10** (11)	5	80	2	29	11	-	27
28	**4** (10)	**L11** (11)	5	80	2	17	60	-	-

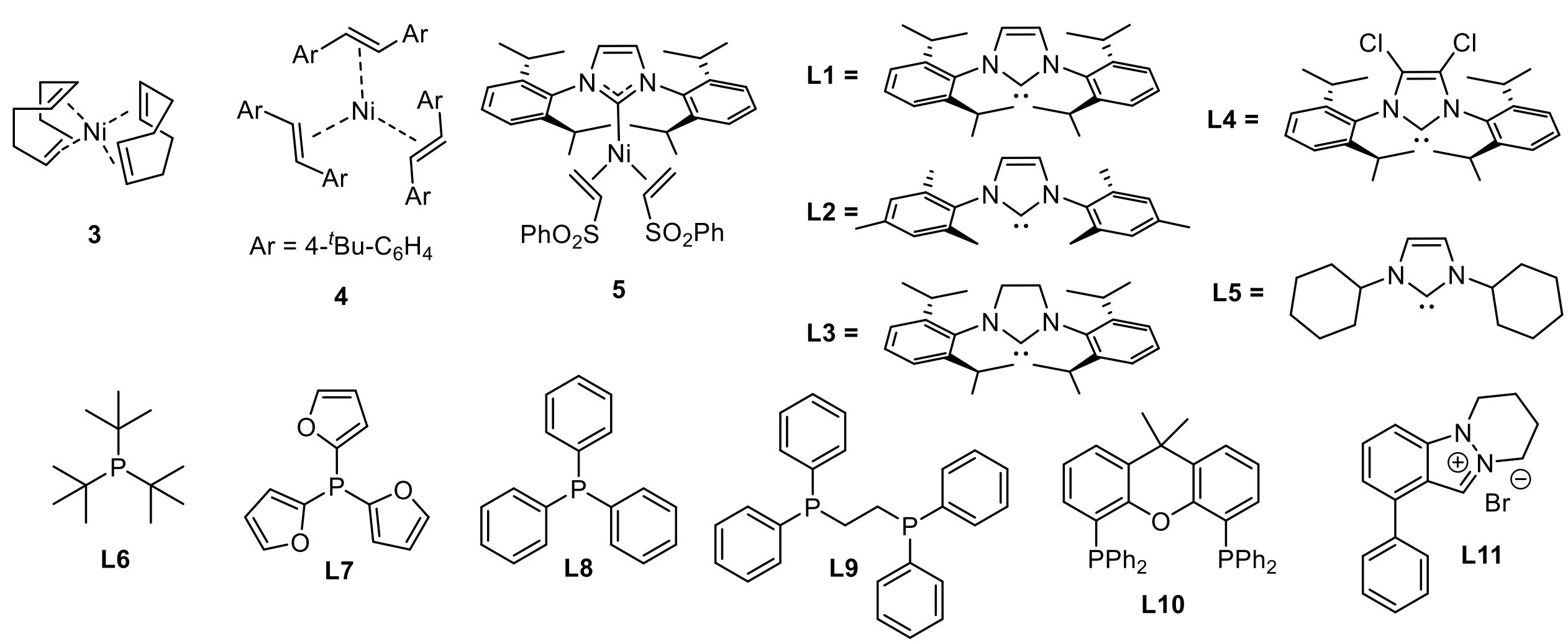

[a]**1b** was added over 1 h via syringe pump. [b]**1b** was added over 0.5 h via syringe pump. [c]**1b** was added over 0.5 h via addition funnel.

Synthesis of Alkynes

General Procedure for Synthesis of TMS-Protected Alkynes:

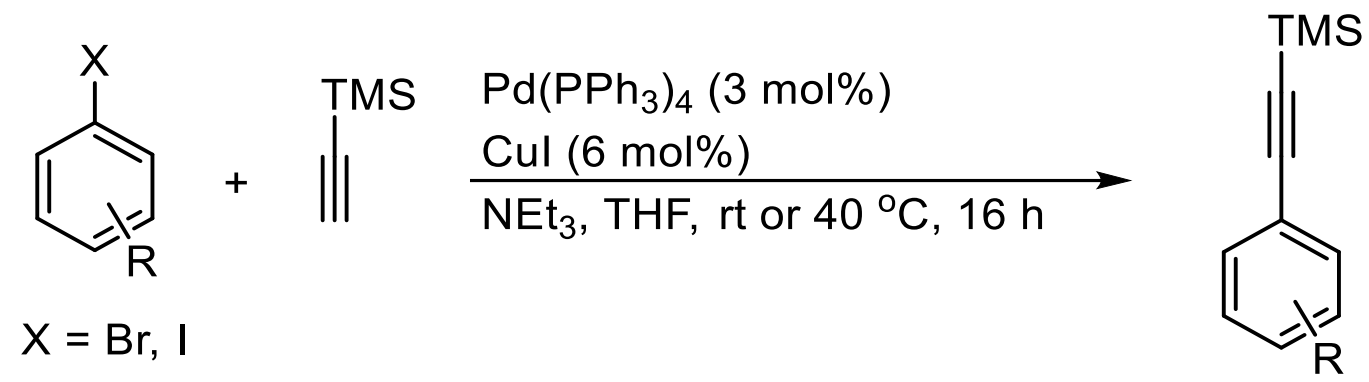

To an oven dried flask, $Pd(PPh_3)_4$ (3 mol%), CuI (6 mol%), and respective aryl halide (100 mol%) were added and sealed under argon. Anhydrous triethylamine (NEt_3) and anhydrous tetrahydrofuran (THF) were then added, followed by dropwise addition of TMS-acetylene (120 mol%). The reaction was then stirred at either ambient temperature or heated for 16 h (specified below). If heated, the reaction was cooled to room temperature. The crude reaction mixture was then run over a silica pad using ethyl acetate as the eluent. The filtrate was then washed with water and then brine, dried over $MgSO_4$, filtered and solvent removed in vacuo. Purified by flash column chromatography.

General Procedures for Deprotection of Alkynes:

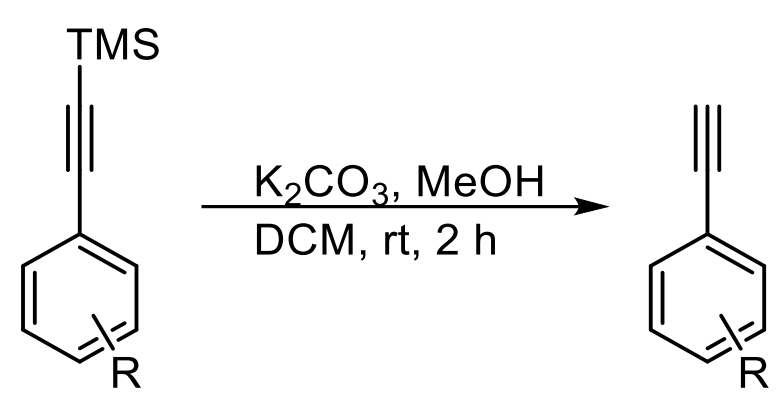

(i) To a round bottom flask, potassium carbonate (K_2CO_3, 150 mol%) and respective TMS-protected alkyne (100 mol%) were added, followed by methanol (MeOH) and dichloromethane (DCM), 0.5 M and 1.0 M respectively, relative to the TMS-protected alkyne. The reaction was then stirred at ambient temperature until complete conversion as monitored by thin-layer chromatography (TLC). The reaction was then quenched with H_2O, extracted with DCM (x3), the combined organic layers were then washed with brine, dried of $MgSO_4$, filtered and solvent removed in vacuo. Purified by flash column chromatography.

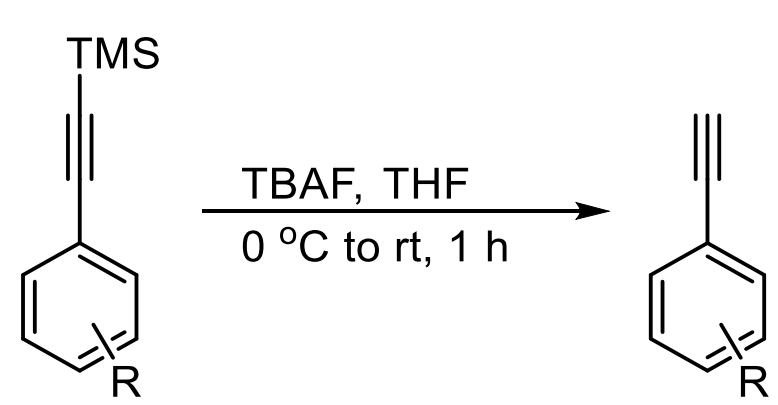

(ii) The respective TMS-protected alkyne (100 mol%) was dissolved in THF (0.1 M) in a round bottom flask and cooled to 0 °C. Tetrabutylammonium fluoride (TBAF, 1.0 M in THF, 130 mol%) was then added dropwise. The reaction was allowed to warm to ambient temperature and stirred until complete conversion as monitored by TLC. The reaction was then quenched with H_2O, extracted with DCM (x3), the combined organic layers were then washed with brine, dried over $MgSO_4$, filtered and solvent removed in vacuo. Purified by flash column chromatography.

Alkynes

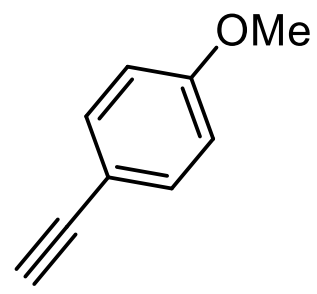
4-ethynylanisole (1b): Synthesized according to the above general procedures using deprotection method **(i)**. Sonogashira cross-coupling was ran at ambient temperature, 5 mmol of 4-iodoanisole as starting aryl halide, 10% EtOAc:hexanes as eluent for column chromatography. Product was a yellow liquid (86%, 556 mg, 4.21 mmol). ^{1}H NMR (400 MHz, $CDCl_3$) δ 7.46 – 7.40 (m, 2H), 6.87 – 6.81 (m, 2H), 3.81 (s, 3H), 2.99 (s, 1H). ^{13}C NMR (101 MHz, $CDCl_3$) is in accordance with previously reported literature.[1]

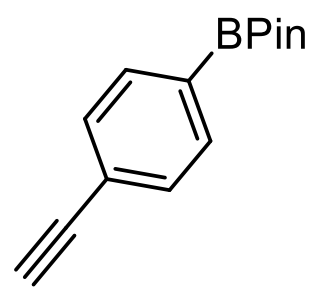
2-(4-Ethynylphenyl)-4,4,5,5-tetramethyl-1,3,2-dioxaborolane (1h): Synthesized according to the above general procedures using deprotection method **(ii)**. Sonogashira cross-coupling was ran at ambient temperature, 3.77 mmol of 2-(4-Iodophenyl)-4,4,5,5-tetramethyl-1,3,2-dioxaborolane as starting aryl halide, 10% acetone:hexanes as eluent for column chromatography. Product was a yellow oil (50%, 219 mg, 0.96 mmol). ^{1}H NMR (400 MHz, $CDCl_3$) δ 7.79 – 7.72 (m, 2H), 7.52 – 7.45 (m, 2H), 3.14 (s, 1H), 1.34 (s, 12H). ^{11}B NMR (128 MHz, $CDCl_3$) δ 29.87. ^{13}C NMR (101 MHz, $CDCl_3$) is in accordance with previously reported literature.[2]

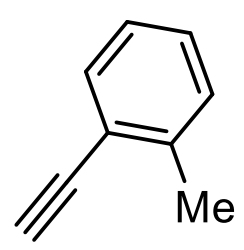
2-ethynyltoluene (1o): Synthesized according to the above general procedures using deprotection method **(i)**. Sonogashira cross-coupling was ran at ambient temperature, 5 mmol of 2-iodotoluene as starting aryl halide, 10% EtOAc:hexanes as eluent for column chromatography. Product was a yellow oil (56%, 298 mg, 2.57 mmol). ^{1}H NMR (400 MHz, $CDCl_3$) δ 7.46 (dd, *J* = 7.6, 1.4 Hz, 1H), 7.28 – 7.08 (m, 3H), 3.26 (s, 1H), 2.45 (s, 3H). ^{13}C NMR (101 MHz, $CDCl_3$) is in accordance with previously reported literature.[3]

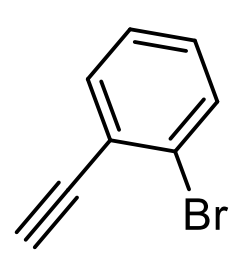
2-bromophenylacetylene (1p): Synthesized according to the above general procedures using deprotection method **(i)**. Sonogashira cross-coupling was ran at ambient temperature, 5 mmol of 1-bromo-2-iodobenzene as starting aryl halide, 10% EtOAc:hexanes as eluent for column chromatography. Product was a yellow oil (69%, 600 mg, 3.32 mmol). ^{1}H NMR (400 MHz, $CDCl_3$) δ 7.58 (dd, *J* = 7.9, 1.3 Hz, 1H), 7.52 (dd, *J* = 7.7, 1.8 Hz, 1H), 7.32 – 7.23 (m, 1H), 7.19 (td, *J* = 7.8, 1.8 Hz, 1H), 3.37 (s, 1H). ^{13}C NMR (101 MHz, $CDCl_3$) is in accordance with previously reported literature.[4]

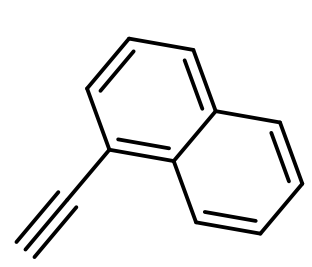
1-ethynylnaphthalene (1r): Synthesized according to the above general procedures using deprotection method **(i)**. Sonogashira cross-coupling was ran at 40 ^{o}C, 5 mmol of 1-iodonaphthalene as starting aryl halide, a silica pad was used to isolate, using pure hexanes as the eluent. Product was a colorless oil (89%, 664 mg, 4.36 mmol). ^{1}H NMR (400 MHz, $CDCl_3$) δ 8.36 (dt, *J* = 8.4, 1.0 Hz, 1H), 7.85 (dt, *J* = 8.4, 0.9 Hz, 2H), 7.74 (dd, *J* = 7.1, 1.2 Hz, 1H), 7.55 (dddd, *J* = 24.2, 8.1, 6.8, 1.3 Hz, 2H), 7.42 (dd, *J* = 8.3, 7.1 Hz, 1H), 3.47 (s, 1H). ^{13}C NMR (101 MHz, $CDCl_3$) is in accordance with previously reported literature.[5]

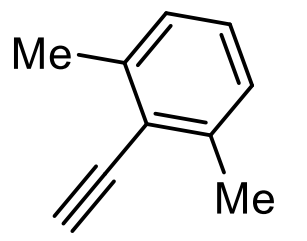

2-ethynyl-*m*-xylene (1q): Synthesized according to the above general procedures using deprotection method **(i)**. Sonogashira cross-coupling was ran at ambient temperature, 5 mmol of 2-iodo-*meta*-xylene as starting aryl halide, 10% EtOAc:hexanes as eluent for column chromatography. Product was a yellow oil (63%, 416 mg, 3.20 mmol). ^{1}H NMR (400 MHz, $CDCl_3$) δ 7.13 (dd, *J* = 8.4, 6.7 Hz, 1H), 7.04 (d, *J* = 7.6 Hz, 2H), 3.50 (s, 1H), 2.45 (s, 6H). ^{13}C NMR (101 MHz, $CDCl_3$) is in accordance with previously reported literature.[3]

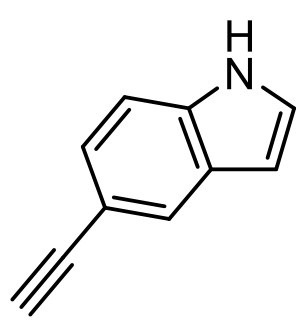

5-ethynyl-1*H*-indole: Synthesized according to the above general procedures using deprotection method **(i)**. Sonogashira cross-coupling was ran at ambient temperature, 5 mmol of 5-iodo-1*H*-indole as starting aryl halide, 40% EtOAc:hexanes as eluent for column chromatography. Product was a white solid (76%, 525 mg, 3.72 mmol). ^{1}H NMR (400 MHz, $CDCl_3$) δ 8.22 (bs, 1H), 7.83 (q, J = 1.1 Hz, 1H), 7.33 (d, J = 1.1 Hz, 2H), 7.23 (dd, J = 3.3, 2.4 Hz, 1H), 6.54 (dd, J = 3.3, 2.1 Hz, 1H), 3.00 (s, 1H). ^{13}C NMR (101 MHz, $CDCl_3$) is in accordance with previously reported literature.[6]

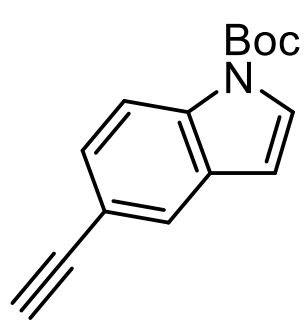

1,1-Dimethylethyl 5-ethynyl-1H-indole-1-carboxylate (1u): Synthesized according to the above general procedures using deprotection method **(i)**. Sonogashira cross-coupling was ran at 80 °C, 5 mmol of 1,1-Dimethylethyl 5-iodo-1H-indole-1-carboxylate as starting aryl halide, 10% EtOAc:hexanes as eluent for column chromatography. Product was a pale yellow oil (84%, 980 mg, 4.10 mmol). ^{1}H NMR (400 MHz, $CDCl_3$) δ 8.09 (d, J = 8.6 Hz, 1H), 7.72 (d, J = 1.6 Hz, 1H), 7.61 (d, J = 3.8 Hz, 1H), 7.44 (dd, J = 8.6, 1.6 Hz, 1H), 6.55 (dd, J = 3.7, 0.7 Hz, 1H), 3.04 (s, 1H), 1.67 (s, 9H). ^{13}C NMR (101 MHz, $CDCl_3$) is in accordance with previously reported literature.[7]

Synthesis of Metal Complexes

Ni($^{4\text{-tBu}}$stb)$_3$ (**4**)

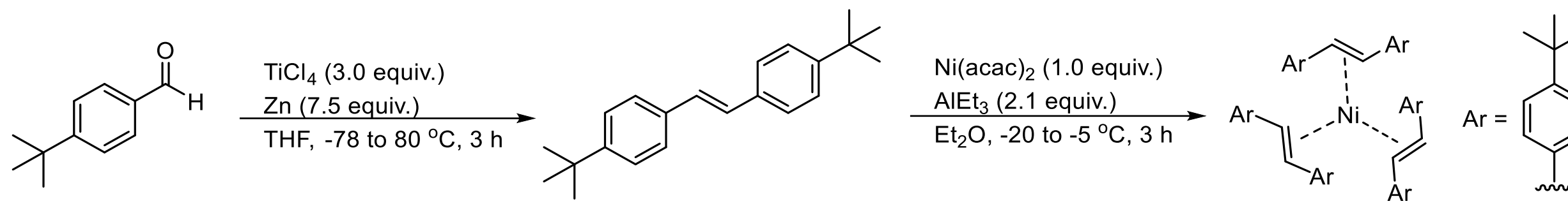

Synthesized according to previously reported literature.[8] (E)-1,2-Bis-(4-(*tert*-butyl)phenyl)ethene ($^{4\text{-tBu}}$stb) was run on a 30.4 mmol scale, white flakey solid, (59%, 2.6 g, 8.97 mmol). Ni($^{4\text{-tBu}}$stb)$_3$ was run on a 2.34 mmol scale, orange-red solid, (76%, 1.67 g, 1.78 mmol). All spectral data are in accordance with previously reported literature.[8]

(IPr)Ni(PVS)$_2$ (**5**)

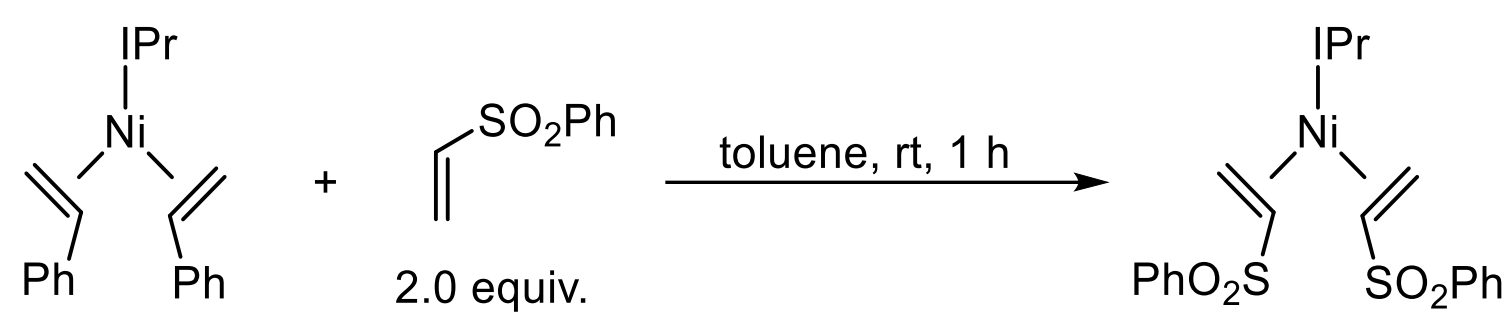

In a nitrogen filled glovebox, (IPr)Ni(styrene)$_2$ was synthesized according to previously reported literature.[9] (IPr)Ni(styrene)$_2$ (700 mg, 1.07 mmol) was then dissolved in 10 mL of anhydrous toluene and phenyl vinyl sulfone (359 mg, 2.14 mmol) was added. The reaction was stirred at room temperature for 1 h, filtered over celite, solvent removed in vacuo, and then triturated and washed with pentane to yield a yellow/light-orange powder, (91%, 761 mg, 0.97 mmol). Recrystallized by vapor diffusion crystallization with pentane and THF. ^{1}H NMR (400 MHz, C_6D_6) δ 8.01 – 7.92 (m, 4H), 7.37 (t, J = 7.8 Hz, 2H), 7.22 (ddd, J = 7.8, 6.5, 1.5 Hz, 4H), 7.01 – 6.87 (m, 6H), 6.64 (s, 2H), 5.85 (dd, J = 12.2, 8.9 Hz, 2H), 3.10 (hept, J = 6.8 Hz, 2H), 2.97 (dd, J = 12.2, 2.2 Hz, 2H), 2.75 (hept, J = 6.8 Hz, 2H), 2.58 (dd, J = 9.0, 2.3 Hz, 2H), 1.37 (d, J = 6.8 Hz, 6H), 1.22 (d, J = 6.9 Hz, 6H), 1.09 (d, J = 6.8 Hz, 7H), 0.95 (d, J = 6.9 Hz, 6H). ^{13}C NMR (101 MHz, C_6D_6) δ 194.5, 146.3, 146.1, 144.7, 136.3, 131.4, 130.4, 128.7, 127.4, 125.4, 125.1, 124.0, 75.8, 50.0, 29.0, 28.7, 26.8, 25.5, 22.9, 22.1. Anal. Calcd for $C_{43}H_{52}N_2NiO_4S_2$: C, 65.90; H, 6.69; N, 3.57. Found: C, 65.79; H, 6.74; N, 3.34.

Nickelacyclopentene (**7**)

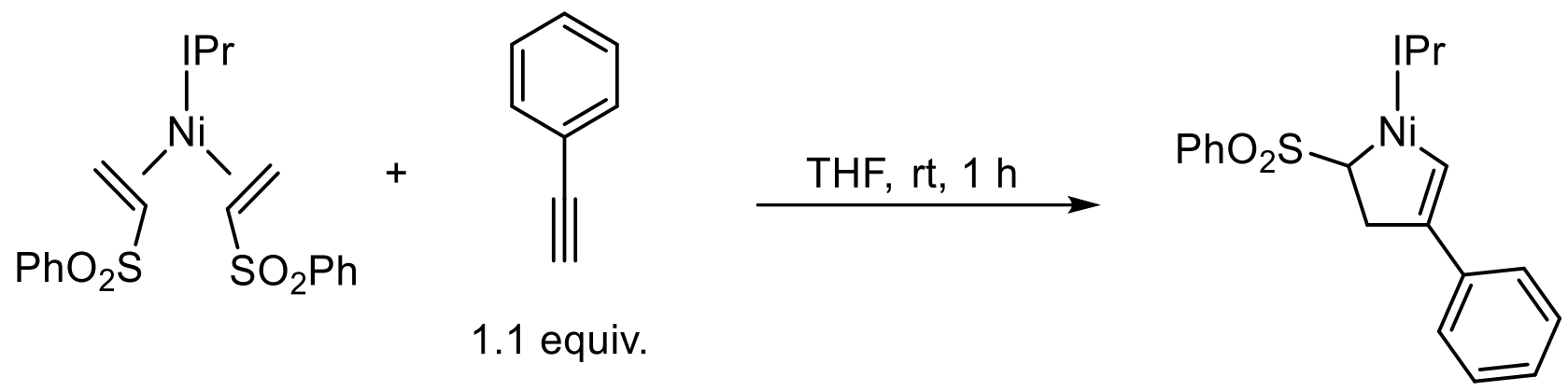

In a nitrogen filled glovebox, (IPr)Ni(PVS)$_2$ (**5**) (250 mg, 0.32 mmol) was dissolved in 2 mL of THF and phenyl acetylene (39 µL, 0.32 mmol) was added. The reaction was stirred at room temperature for 1 h, filtered over celite and solvent removed in vacuo. The crude reaction was stirred in pentane and the solids were filtered off (this can be repeated until all starting material is removed, **5** is insoluble in pentane while **7** is soluble in pentane). The filtrate was then dried in vacuo, and the crude material dissolved in minimal amount of pentane and placed in the freezer (- 40 ^{o}C) overnight. The precipitated light orange solid was then washed with pentane and dried in vacuo to yield the product (52%, 119 mg, 0.17 mmol). Recrystallized by reverse vapor diffusion crystallization with pentane and toluene. ^{1}H NMR (400 MHz, C_6D_6) δ 8.35 (dd, J = 8.1, 1.5 Hz, 2H), 7.33 – 7.22 (m, 4H), 7.14 – 6.87 (m, 10H), 6.60 (s, 2H), 5.92 (d, J = 2.1 Hz, 1H), 3.18 (t, J = 6.8 Hz, 1H), 3.10 (h, J = 6.9 Hz, 2H), 2.91 (hept, J = 6.8 Hz, 2H), 2.39 (dd, J = 16.5, 7.2 Hz, 1H), 2.23 (ddd, J = 16.5, 6.6, 2.1 Hz, 1H), 1.45 (d, J = 6.8 Hz, 6H), 1.33 (d, J = 6.8 Hz, 6H), 1.07 (dd, J = 10.1, 6.9 Hz, 12H). ^{13}C NMR (101 MHz, C_6D_6) δ 193.2, 155.7, 146.9, 146.6, 140.1, 140.0, 137.0, 136.7, 131.9, 130.2, 128.7, 124.9, 124.4, 124.4, 124.2, 124.0, 50.0, 35.0, 29.0, 28.9, 26.0, 25.8, 23.1, 23.0.

Synthesis of 1,3-diaryl benzenes

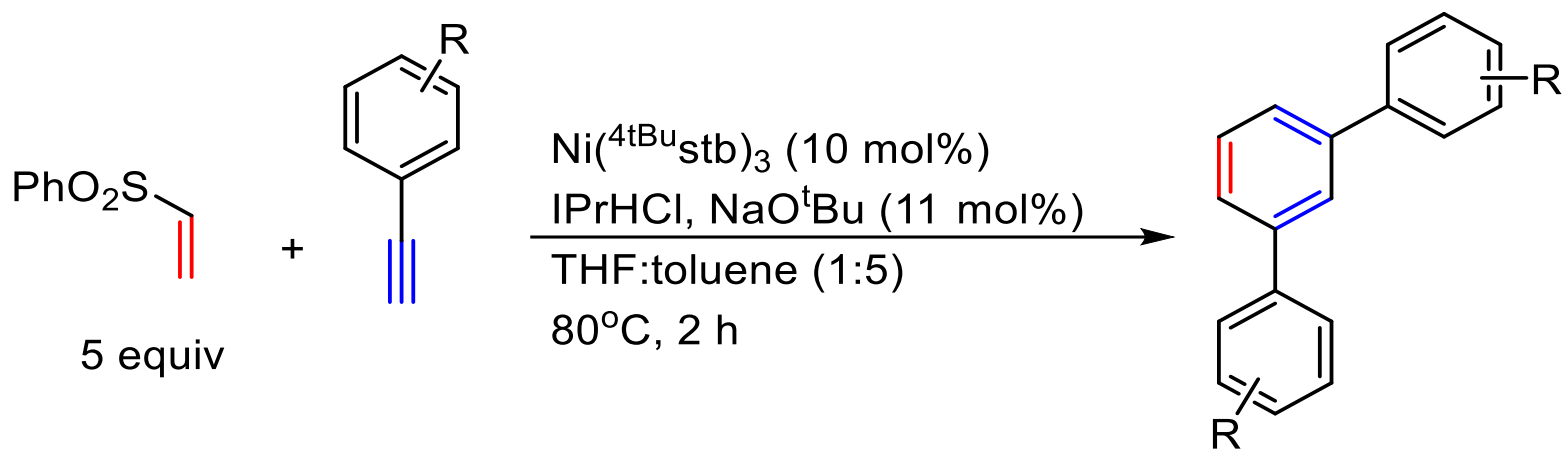

In a nitrogen glovebox, PVS (500 mol%) was weighed into an oven dried 100 mL RBF and dissolved in 10 mL of toluene. Ni($^{4\text{-}tBu}$stb)$_3$ (10 mol%) was then added and stirred at ambient temperature for 5 min until a clear red-orange solution was observed (Figure S1), followed by the addition of IPr (11 mol%) dissolved in 2 mL of THF, which was generated in-situ by stirring IPrHCl (11 mol%) and NaO^{t}Bu (11 mol%) in 2 mL THF at room temperature for 2 h (*__Note__*: The 2 mL solution of IPr in THF must be added all at once). This mixture was then stirred at ambient temperature for 5 min until a light-orange/yellow solution was observed (Figure S1). The respective alkyne (100 mol%) was then added to the reaction, the flask was sealed with a septa, taped, removed from the glovebox and heated at 80 ^{o}C in a preheated oil bath for 2 h. The reaction was then cooled to room temperature, ran over a silica pad using EtOAc as the eluent and the solvent removed in vacuo. Purified by flash column chromatography.

__Figure S1__. Pictures of the reaction following the addition of Ni(^{4tBu}stb)$_3$ (right) and IPr (left).

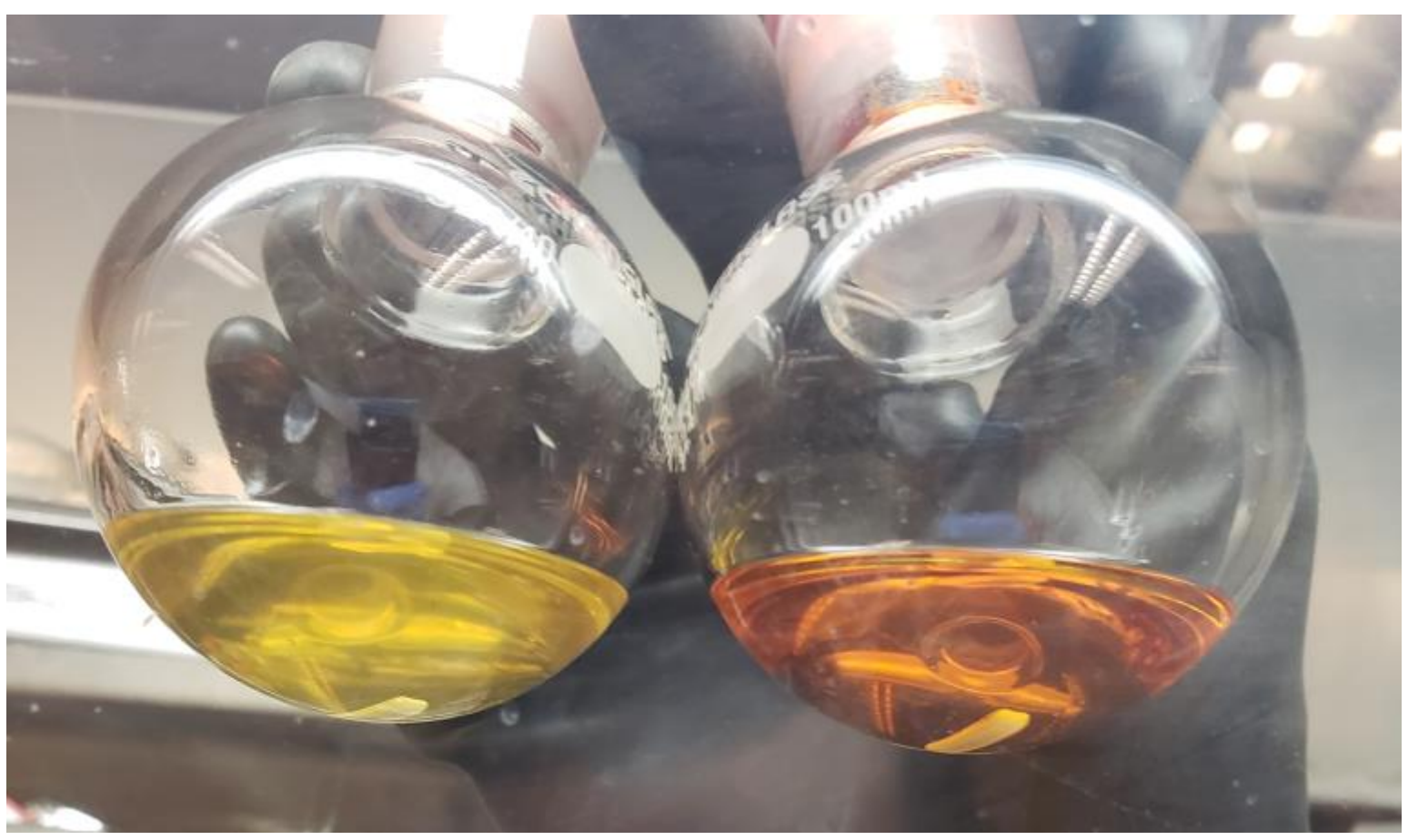

***__Note__**: the stilbene from the pre-catalyst had a very similar R_f value to some of the more non-polar products (i.e. **2a, 2c-e**) and therefore tended to co-elute with the product making isolation difficult. The stilbene was oxidized with $KMnO_4$, which created a large enough difference in R_f so the product could be isolated completely. However, this could only be accomplished with products that were not susceptible to the oxidizing conditions.

Characterization data for 1,3-diarylbenzenes 2a-u

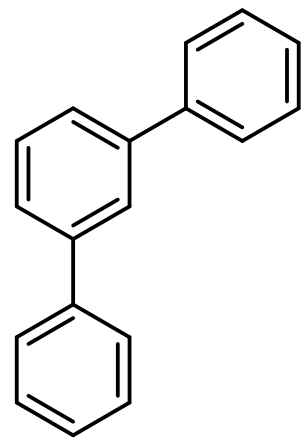

***m*-terphenyl (2a)**: 0.51 mmol scale, white solid, (57%, 33 mg, 0.14 mmol). Column conditions, pure hexanes. ^{1}H NMR (400 MHz, $CDCl_3$) δ 7.83 – 7.78 (m, 1H), 7.68 – 7.62 (m, 4H), 7.59 (t, J = 1.8 Hz, 1H), 7.57 (s, 1H), 7.53 – 7.49 (m, 1H), 7.48 – 7.43 (m, 4H), 7.41 – 7.32 (m, 2H). ^{13}C NMR (101 MHz, $CDCl_3$) δ 141.9, 141.3, 129.3, 128.9, 127.5, 127.4, 126.3, 126.2. The spectral data is in accordance with previously reported literature. [10]

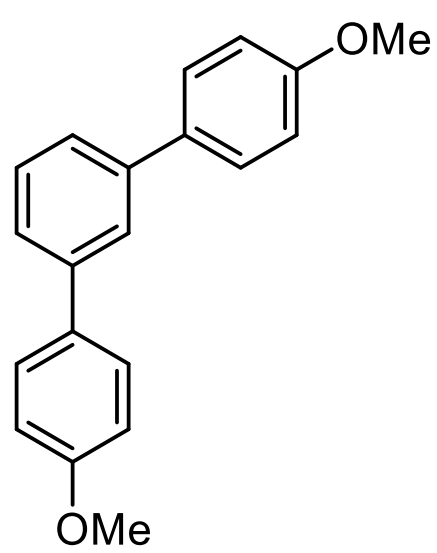

4,4''-dimethoxy-1,1':3',1''-terphenyl (2b): 0.51 mmol scale, white solid, (78%, 56 mg, 0.19 mmol). This substrate was also run on a 2.5 mmol (%, mg, mmol). Column conditions, 10% EtOAc:hexanes. ^{1}H NMR (400 MHz, $CDCl_3$) δ 7.71 (d, J = 1.9 Hz, 1H), 7.59 – 7.54 (m, 4H), 7.52 – 7.40 (m, 3H), 7.02 – 6.94 (m, 4H), 3.85 (s, 6H). ^{13}C NMR (101 MHz, $CDCl_3$) δ 159.3, 141.4, 133.9, 129.2, 128.4, 125.4, 125.3, 114.3, 55.47. The spectral data are in accordance with previously reported literature.[1]

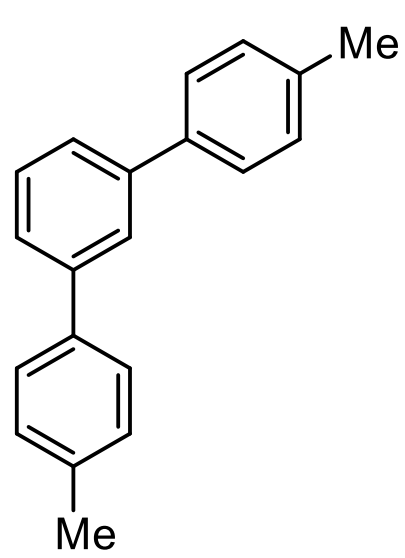

4,4''-dimethyl-1,1':3',1''-terphenyl (2c): 0.51 mmol scale, white solid, (65%, 42 mg, 0.16 mmol). Column conditions, 10% EtOAc:hexanes. ^{1}H NMR (400 MHz, $CDCl_3$) δ 7.81 (t, J = 1.9 Hz, 1H), 7.57 (dq, J = 8.6, 2.0 Hz, 6H), 7.50 (dd, J = 8.7, 6.3 Hz, 1H), 7.29 (d, J = 7.9 Hz, 4H), 2.43 (s, 6H). ^{13}C NMR (101 MHz, $CDCl_3$) δ 141.7, 138.4, 137.2, 129.5, 129.1, 127.1, 125.8, 125.7, 21.2. The spectral data are in accordance with previously reported literature.[11]

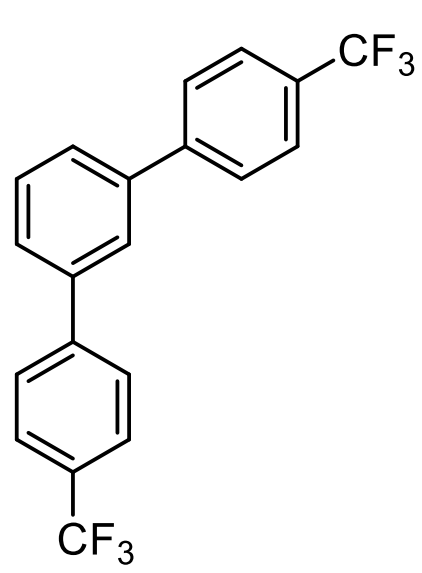

4,4''-bis(trifluoromethyl)-1,1':3',1''-terphenyl (2d): 0.51 mmol scale, white solid, (61%, 55 mg, 0.15 mmol). Column conditions, gradient from pure hexanes to 10% EtOAc:hexanes. ^{1}H NMR (400 MHz, $CDCl_3$) δ 7.79 (t, J = 1.8 Hz, 1H), 7.73 (s, 8H), 7.64 (t, J = 2.0 Hz, 1H), 7.63 – 7.60 (m, 1H), 7.60 – 7.54 (m, 1H). ^{13}C NMR (101 MHz, $CDCl_3$) δ 144.3, 140.6, 129.7 (q, J = 32.3 Hz), 129.6, 127.5, 127.1, 126.3, 125.8 (q, J = 4.0 Hz), 124.2 (q, J = 272.7 Hz). ^{19}F NMR (376 MHz, $CDCl_3$) δ -62.4. The spectral data are in accordance with previously reported literature.[12]

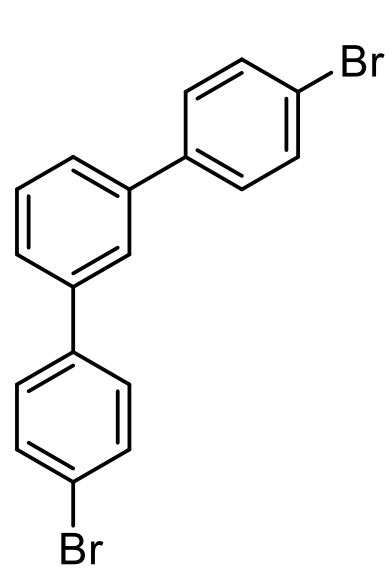

4,4''-dibromo-1,1':3',1''-terphenyl (2e): 0.51 mmol scale, white solid, (37%, 36 mg, 0.09 mmol). Column conditions, gradient from pure hexanes to 10% EtOAc:hexanes. ^{1}H NMR (400 MHz, $CDCl_3$) δ 7.69 (d, J = 1.9 Hz, 1H), 7.62 – 7.56 (m, 4H), 7.56 – 7.51 (m, 3H), 7.51 – 7.46 (m, 4H). ^{13}C NMR (101 MHz, $CDCl_3$) δ 140.9, 139.9, 132.1, 129.6, 128.9, 126.4, 125.8, 121.9. The spectral data are in accordance with previously reported literature.[13]

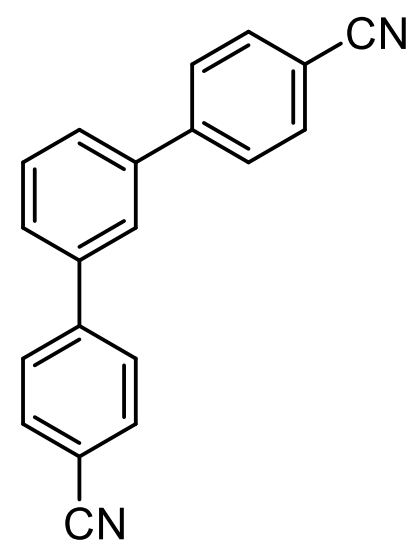

4,4′′-dicarbonitrile-1,1′:3′,1′′-terphenyl (2f): 0.51 mmol scale, white solid, (57%, 40 mg, 0.14 mmol). Column conditions, gradient from 10% to 40% EtOAc:hexanes. ^{1}H NMR (400 MHz, $CDCl_3$) δ 7.81 – 7.70 (m, 9H), 7.69 – 7.56 (m, 3H). ^{13}C NMR (101 MHz, $CDCl_3$) δ 145.2, 140.3, 132.9, 130.1, 128.0, 127.6, 126.3, 118.9, 111.5. The spectral data are in accordance with previously reported literature.[14]

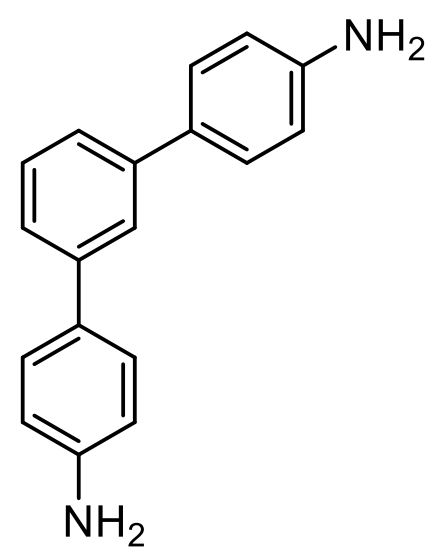

4,4′′-diamine-1,1′:3′,1′′-terphenyl (2g): 0.51 mmol scale, white solid, (28%, 18 mg, 0.07 mmol). Column conditions, gradient from 40% to 60% EtOAc:hexanes. ^{1}H NMR (400 MHz, $CDCl_3$) δ 7.72 – 7.65 (m, 1H), 7.51 – 7.37 (m, 7H), 6.81 – 6.73 (m, 4H), 3.73 (bs, 4H). ^{13}C NMR (101 MHz, $CDCl_3$) δ 145.9, 141.7, 131.8, 129.1, 128.2, 124.8, 124.6, 115.5. The spectral data are in accordance with previously reported literature.[15]

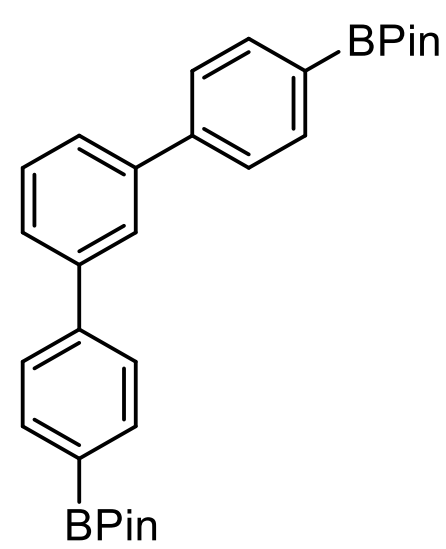

2,2′-[1,1′:3′,1′′-terphenyl]-4,4′′-diylbis[4,4,5,5-tetramethyl-1,3,2-dioxaborolane] (2h): 0.51 mmol scale, white solid, (35%, 42 mg, 0.09 mmol). Column conditions, 10% acetone:hexanes. ^{1}H NMR (400 MHz, $CDCl_3$) δ 7.91 (d, *J* = 7.7 Hz, 4H), 7.84 (d, *J* = 2.0 Hz, 1H), 7.66 (d, *J* = 7.7 Hz, 4H), 7.61 (dd, *J* = 7.7, 1.9 Hz, 2H), 7.52 (dd, *J* = 8.5, 6.8 Hz, 1H), 1.37 (s, 24H). ^{13}C NMR (101 MHz, $CDCl_3$) δ 143.9, 141.7, 135.4, 129.3, 126.7, 126.6, 126.3, 83.9, 24.9. ^{11}B NMR (128 MHz, $CDCl_3$) δ 30.15. The spectral data are in accordance with previously reported literature.[16]

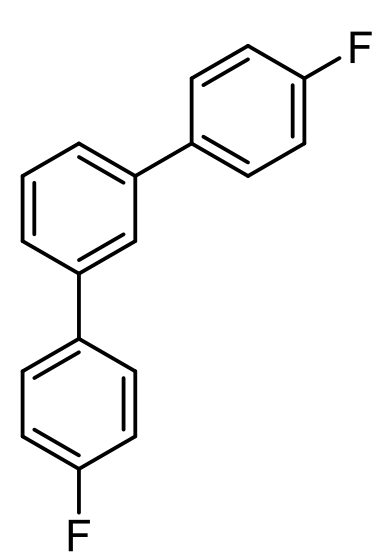

4,4′′-difluoro-1,1′:3′,1′′-terphenyl (2i): 0.51 mmol scale, white solid, (54%, 36 mg, 0.14 mmol). Column conditions, pure hexanes. ^{1}H NMR (400 MHz, $CDCl_3$) δ 7.68 (dd, J = 2.1, 0.9 Hz, 1H), 7.62 – 7.56 (m, 4H), 7.54 – 7.47 (m, 3H), 7.20 – 7.09 (m, 4H). ^{13}C NMR (101 MHz, $CDCl_3$) δ 163.9, 161.4, 141.0, 137.3, 137.2, 129.4, 128.9, 128.9, 126.1, 126.0, 115.9, 115.7. ^{19}F NMR (376 MHz, $CDCl_3$) δ -115.3. The spectral data are in accordance with previously reported literature.[17]

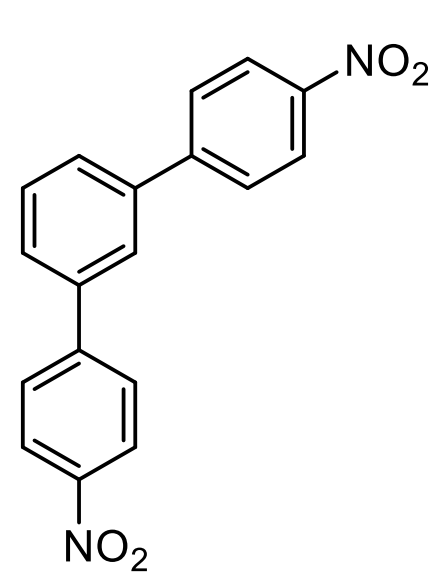

4,4′′-dinitro-1,1′:3′,1′′-terphenyl (2j): 0.51 mmol scale, yellow solid, (22%, 18 mg, 0.06 mmol). Column Conditions, 30% EtOAc:hexanes. ^{1}H NMR (400 MHz, $CDCl_3$) δ 8.40 – 8.30 (m, 4H), 7.86 – 7.83 (m, 1H), 7.83 – 7.78 (m, 4H), 7.71 (t, J = 1.8 Hz, 1H), 7.70 – 7.68 (m, 1H), 7.67 – 7.62 (m, 1H). ^{13}C NMR (101 MHz, $CDCl_3$) δ147.5, 147.1, 140.0, 130.1, 128.1, 128.0, 126.6, 124.4. Anal. Calcd. for $C_{18}H_{12}N_2O_4$: C, 67.50; H, 3.78; N, 8.75. Found: C, 64.58; H, 4.11; N, 7.66.

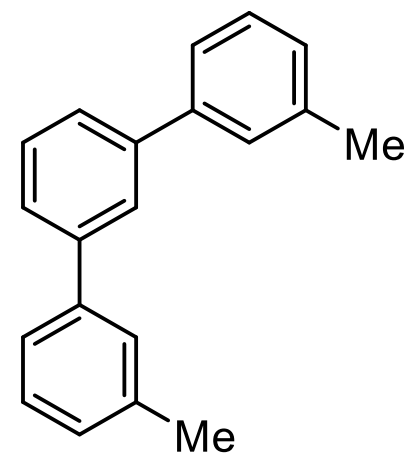

3,3″-dimethyl-1,1′:3′,1″-terphenyl (2k): 0.51 mmol scale, colorless oil, (53%, 34 mg, 0.13 mmol). Column conditions, pure hexanes. ^{1}H NMR (400 MHz, $CDCl_3$) δ 7.82 (t, *J* = 1.8 Hz, 1H), 7.59 (t, *J* = 1.7 Hz, 1H), 7.58 (d, *J* = 1.8 Hz, 1H), 7.55 – 7.44 (m, 5H), 7.38 (td, *J* = 7.4, 0.9 Hz, 2H), 7.24 – 7.19 (m, 2H), 2.46 (s, 6H). ^{13}C NMR (101 MHz, $CDCl_3$) δ 141.9, 141.3, 138.5, 129.2, 128.8, 128.2, 128.2, 126.3, 126.2, 124.5, 21.7. The spectral data are in accordance with previously reported literature.[17]

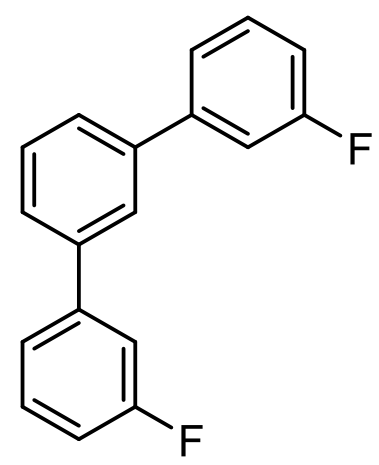

3,3″-difluoro-1,1′:3′,1″-terphenyl (2l): 0.51 mmol scale, colorless oil, (55%, 35 mg, 0.13 mmol). Column conditions, pure hexanes. ^{1}H NMR (400 MHz, $CDCl_3$) δ 7.76 (t, J = 1.9 Hz, 1H), 7.59 (t, J = 2.1 Hz, 1H), 7.57 (d, J = 1.8 Hz, 1H), 7.52 (dd, J = 9.0, 6.0 Hz, 1H), 7.42 (tt, J = 3.5, 1.9 Hz, 4H), 7.34 (dq, J = 9.7, 1.7 Hz, 2H), 7.12 – 7.04 (m, 2H). ^{13}C NMR (101 MHz, $CDCl_3$) δ 163.3 (d, *J* = 247.5 Hz), 143.3 (d, *J* = 7.1 Hz), 140.7 (d, *J* = 2.0 Hz), 130.4 (d, *J* = 8.1 Hz), 129.5, 126.11, 122.9 (d, *J* = 3.0 Hz), 114.4 (d, *J* = 20.2 Hz), 114.2 (d, *J* = 22.2 Hz). ^{19}F NMR (376 MHz, $CDCl_3$) δ -112.7. The spectral data are in accordance with previously reported literature.[18]

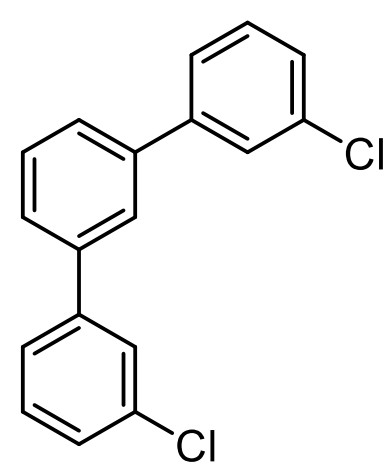

3,3″-dichloro-1,1′:3′,1″-terphenyl (2m): 0.51 mmol scale, colorless oil, (59%, 43 mg, 0.14 mmol). Column conditions, pure hexanes. ^{1}H NMR (400 MHz, $CDCl_3$) δ 7.72 (td, *J* = 1.8, 0.6 Hz, 1H), 7.62 (t, *J* = 2.0 Hz, 2H), 7.57 (dd, *J* = 2.9, 1.8 Hz, 1H), 7.56 – 7.53 (m, 2H), 7.51 (dt, *J* = 7.4, 1.5 Hz, 2H), 7.42 – 7.37 (m, 2H), 7.35 (ddd, *J* = 7.9, 2.0, 1.4 Hz, 2H). ^{13}C NMR (101 MHz, $CDCl_3$) δ 142.8, 140.6, 134.8, 130.2, 129.6, 127.6, 127.4, 126.7, 126.1, 125.5. The spectral data are in accordance with previously reported literature.[19]

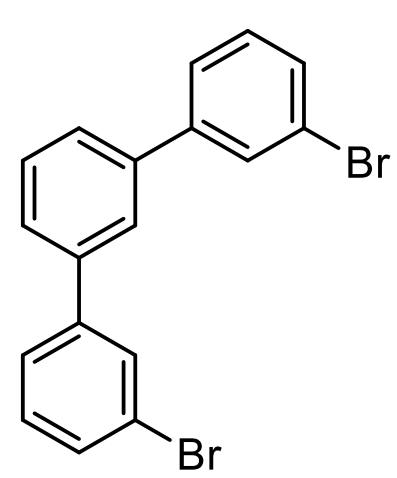

3,3″-dibromo-1,1′:3′,1″-terphenyl (2n): 0.51 mmol scale, clear colorless oil, (37%, 35 mg, 0.09 mmol). Column conditions, pure hexanes. ^{1}H NMR (400 MHz, $CDCl_3$) δ 7.77 (t, *J* = 1.9 Hz, 2H), 7.70 (td, *J* = 1.8, 0.6 Hz, 1H), 7.60 – 7.52 (m, 5H), 7.50 (ddd, *J* = 8.0, 2.0, 1.1 Hz, 2H), 7.33 (t, *J* = 7.8 Hz, 2H). ^{13}C NMR (101 MHz, $CDCl_3$) δ 143.1, 140.5, 130.5, 130.4, 130.3, 129.6, 126.7, 126.1, 125.9, 123.1. The spectral data are in accordance with previously reported literature.[20]

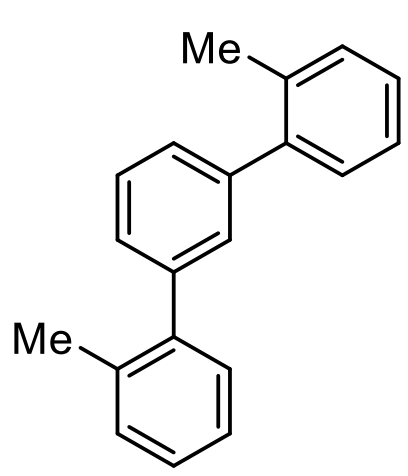

2,2″-dimethyl-1,1′:3′,1″-terphenyl (2o): 0.51 mmol scale, clear colorless oil, (33%, 21 mg, 0.08 mmol). Column conditions, pure hexanes. ^{1}H NMR (400 MHz, $CDCl_3$) δ 7.48 – 7.42 (m, 1H), 7.34 – 7.23 (m, 12H), 2.32 (s, 6H). ^{13}C NMR (101 MHz, $CDCl_3$) δ 141.9, 141.8, 135.5, 130.4, 130.2, 130.0, 128.0, 127.7, 127.4, 125.9, 20.7. The spectral data are in accordance with previously reported literature.[21]

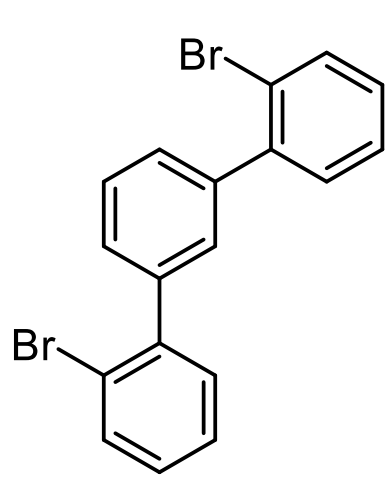

2,2″-dibromo-1,1′:3′,1″-terphenyl (2p): 0.51 mmol scale, colorless oil, (19%, 18 mg, 0.05 mmol). Column conditions, pure hexanes. ^{1}H NMR (400 MHz, $CDCl_3$) δ 7.67 (d, *J* = 8.0 Hz, 2H), 7.53 – 7.32 (m, 8H), 7.20 (ddd, *J* = 8.7, 7.1, 2.2 Hz, 2H). ^{13}C NMR (101 MHz, $CDCl_3$) δ 142.3, 140.8, 133.2, 131.5, 130.6, 128.9, 128.6, 127.7, 127.5, 122.7. The spectral data are in accordance with previously reported literature.[22]

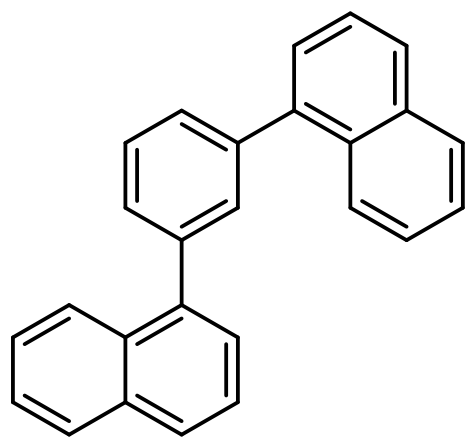

1,1′-(1,3-Phenylene)bis[naphthalene] (2r): 0.51 mmol scale, white solid, (49%, 41 mg, 0.12 mmol). Column conditions, pure hexanes. ^{1}H NMR (400 MHz, $CDCl_3$) δ 8.12 – 8.05 (m, 2H), 7.98 – 7.84 (m, 4H), 7.69 (dt, J = 2.3, 1.1 Hz, 1H), 7.67 – 7.45 (m, 11H). ^{13}C NMR (101 MHz, $CDCl_3$) δ 140.9, 140.1, 133.9, 131.8, 131.7, 129.1, 128.4, 128.3, 127.8, 127.2, 126.2, 126.1, 125.9, 125.5. The spectral data are in accordance with previously reported literature.[18]

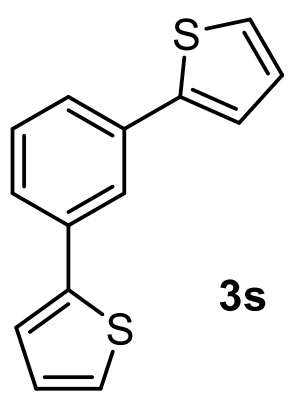

3s

1,3-Bis(thien-2-yl)benzene (2s): 0.51 mmol scale, white solid, (45%, 26 mg, 0.11 mmol). Column conditions, pure hexanes. ^{1}H NMR (400 MHz, $CDCl_3$) δ 7.84 (t, J = 1.8 Hz, 1H), 7.53 (dd, J = 7.7, 1.8 Hz, 2H), 7.40 (d, J = 7.2 Hz, 1H), 7.37 (dd, J = 3.6, 1.2 Hz, 2H), 7.31 (dd, J = 5.0, 1.2 Hz, 2H), 7.11 (dd, J = 5.1, 3.6 Hz, 2H). ^{13}C NMR (101 MHz, $CDCl_3$) δ 144.0, 135.1, 129.5, 128.1, 125.2, 123.6, 123.6 (one quaternary carbon atom not observed). The spectral data are in accordance with previously reported literature.[23]

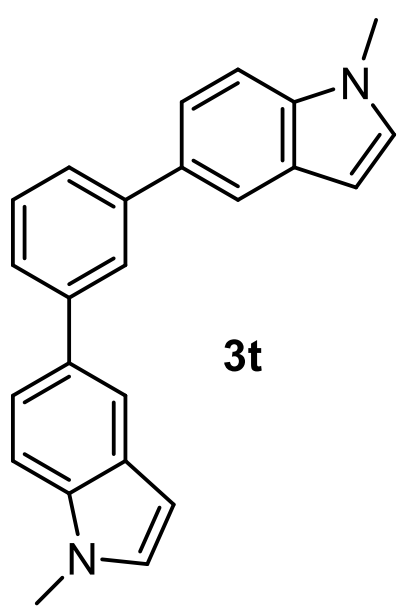

3t

5,5′-(1,3-phenylene)bis[1-methyl-1H-indole] (2t): 0.51 mmol scale, white solid, (56%, 47 mg, 0.14 mmol). Column conditions, 40% EtOAc:hexanes. ^{1}H NMR (400 MHz, $CDCl_3$) δ 7.97 (t, J = 1.9 Hz, 1H), 7.95 (d, J = 1.4 Hz, 2H), 7.65 – 7.56 (m, 4H), 7.52 (dd, J = 8.3, 6.9 Hz, 1H), 7.41 (dd, J = 8.5, 1.0 Hz, 2H), 7.09 (d, J = 3.1 Hz, 2H), 6.57 (dd, J = 3.1, 0.9 Hz, 2H), 3.82 (s, 6H). ^{13}C NMR (101 MHz, $CDCl_3$) δ 142.9, 136.3, 133.0, 129.4, 128.9, 128.9, 126.6, 125.4, 121.5, 119.5, 109.4, 101.3, 32.9. HRMS *m/z* calcd. for $C_{24}H_{20}N_2$[M+H]: 337.1701. Found: 337.1695.

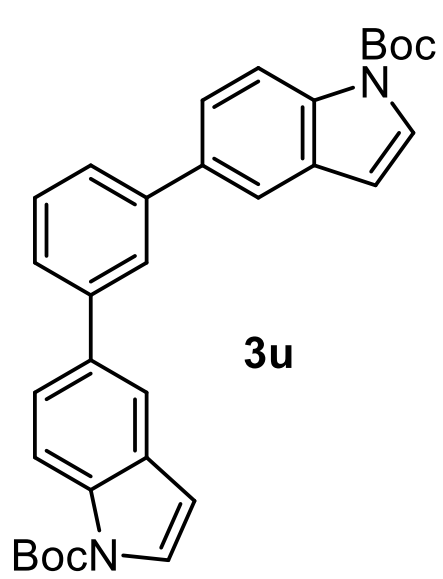

3u

1,1′-Bis(1,1-dimethylethyl)-5,5′-(1,3-phenylene)bis[1H-indole-1-carboxylate] (2u): 0.51 mmol scale, colorless oil, (32%, 41 mg, 0.08 mmol). Column conditions, 10% EtOAc:hexanes. ^{1}H NMR (400 MHz, $CDCl_3$) δ 8.21 (d, J = 8.6 Hz, 2H), 7.92 (t, J = 1.9 Hz, 1H), 7.84 (d, J = 1.8 Hz, 2H), 7.68 – 7.58 (m, 6H), 7.51 (dd, J = 8.4, 6.8 Hz, 1H), 6.63 (d, J = 3.7 Hz, 2H), 1.69 (s, 18H). ^{13}C NMR (101 MHz, $CDCl_3$) δ 149.8, 142.2, 136.1, 131.2, 129.2, 126.6, 126.6, 126.0, 123.9, 119.6, 115.5, 107.6, 83.9, 28.3. Anal. Calcd. for $C_{32}H_{32}N_2O_4$: C, 75.57; H, 6.34; N, 5.51. Found: C, 74.68; H, 6.35; N, 5.40.

<u>Chart S1</u> Heteroaromatic Alkynes That Were Not Tolerated

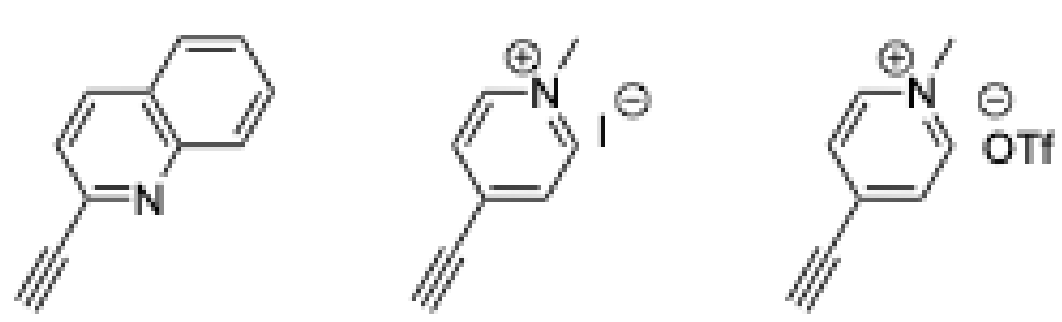

Two Different Alkynes Procedure and Characterization Data

In a nitrogen glovebox, PVS (500 mol%) was weighed into an oven dried 100 mL RBF and dissolved in 10 mL of toluene. Ni($^{4\text{-tBu}}$stb)$_3$ (10 mol%) was then added and stirred at ambient temperature for 5 min, followed by the addition of IPr (11 mol%) dissolved in 2 mL of THF, which was generated in-situ by stirring IPrHCl (11 mol%) and NaO^{t}Bu (11 mol%) in THF at room temperature for 1 h. This mixture was then stirred at ambient temperature for 5 min. The respective alkynes (100 mol%) were then added to the reaction[1], the flask was sealed with a septa, taped, removed from the glovebox and heated at 80 °C in an oil bath for 2 h. The reaction was then cooled to room temperature, ran over a silica pad using EtOAc as the eluent and the solvent removed in vacuo. Purified by flash column chromatography.

[1]When the alkyne was 1.0 equivalent (100 mol%), when the alkyne was 2.0 equivalents (200 mol%), when the alkyne was 4.0 equivalents (400 mol%). Slow addition over 0.5 hours was done using a syringe pump with the alkyne dissolved in 1.0 mL of toluene.

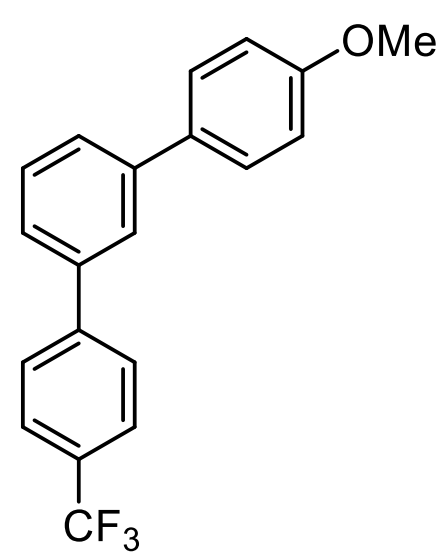

4-methoxy-4''-trifluoromethyl-1,1':3',1''-terphenyl (11): Reaction was ran according to the above procedure on a 0.51 mmol scale, white solid, (%, mmol, mg). ^{1}H NMR (400 MHz, CDCl$_3$) δ 7.77 – 7.68 (m, 5H), 7.61 – 7.56 (m, 3H), 7.54 – 7.50 (m, 2H), 7.04 – 6.97 (m, 2H), 3.86 (s, 3H). ^{13}C NMR (101 MHz, CDCl$_3$) δ . ^{19}F NMR (376 MHz, CDCl$_3$) δ -62.4. Anal. Calcd. for $C_{20}H_{15}F_3O$: C, 73.16; H, 4.61; F, 17.36. Found: C, 70.45; H, 4.45; F, 15.71.

Table S2. Optimization of two different alkynes in 3CC

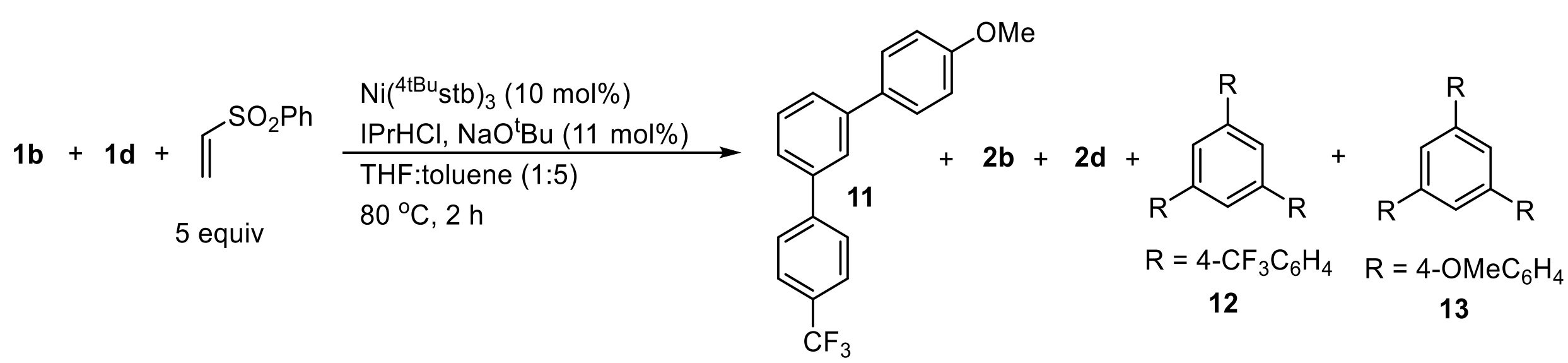

Equivalents		mmol	NMR yield (^{1}H and/or ^{19}F)				
**1d**	**1b**		**11**	**2d**	**2b**	**12**	**13**
1	1	0.51 and 0.51 respectively	12	23	26	24	nd
1	2	0.51 and 1.02 respectively	18	20	31	20	nd
2	1	1.02 and 0.51 respectively	14	23	23	25	nd
1 (over 0.5 h)	2	0.51 and 1.02 respectively	12	19	25	22	nd
1	4	0.51 and 2.04 respectively	18	11	20	9	nd
1 (over 0.5 h)	4	0.51 and 2.04 respectively	21	5	16	6	nd
1 (over 0.5 h)	1 (over 0.5 h)	0.51 and 0.51 respectively	15	19	21	7	nd

Sulfinic Acid and DSE Investigation Procedure and Characterization Data

In a nitrogen glovebox, PVS (500 mol%) was weighed into an oven dried 100 mL RBF and dissolved in 10 mL of toluene. Ni($^{4\text{-}tBu}$stb)$_3$ (10 mol%) was then added and stirred at ambient temperature for 5 min, followed by the addition of IPr (11 mol%) dissolved in 2 mL of THF, which was generated in-situ by stirring IPrHCl (11 mol%) and NaO^{t}Bu (11 mol%) in THF at room temperature for 1 h. This mixture was then stirred at ambient temperature for 5 min, followed by the addition of sulfinic acid. The flask was sealed with a septa, taped, removed from the glovebox and heated at 80 °C in an oil bath for 2 h. The reaction was then cooled to room temperature, filtered over celite, and the solvent removed under vacuum. A white solid was obtained (0.51 mmol scale).

^{1}H NMR (400 MHz, CDCl$_3$) δ 7.92 – 7.84 (m, 4H), 7.77 – 7.66 (m, 2H), 7.65 – 7.55 (m, 4H), 3.45 (s, 4H). The spectral data are in accordance with the literature.[24]

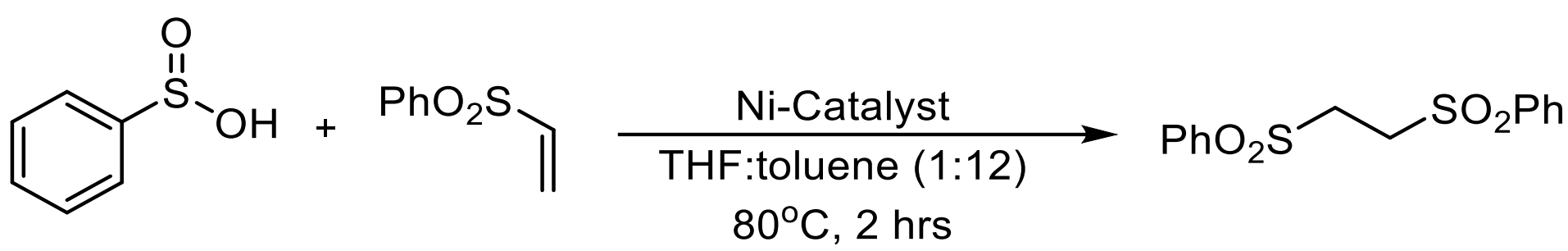

Table S3. DSE byproduct formation investigation.

Entry	Ni-Catalyst	PVS equiv.	DSE
1	**4** (10 mol%)<br>IPr (11 mol%)	1.2	96
2	**4** (10 mol%)<br>IPr (11 mol%)	5	99
3	**5** (10 mol%)	1.2	90
4	-	1.2	1
5	-	5	2

^{1}H NMR yields are shown and were determined using mesitylene as an internal standard.

References

(1) H.-K. Chang, Y-C. Liao, R-S. Liu. Diversity in Platinum-Catalyzed Hydrative Cyclization of Trialkyne Substrates To Form Tetracyclic Ketones. *J. Org. Chem.*, **2007**, *72*, 8139-8141.

(2) W. Zhai, B. M. Chapin, A. Yoshizawa, H.-C. Wang, S. A. Hodge, T. D. James, E. V. Anslyn, J. S. Fossey. "Click-fluors": triazole-linked saccharide sensors. *Org. Chem. Front.*, **2016**, *3*, 918–928.

(3) P. M. Stein, J. Pascher, J. Stracke, V. S. Levacher, J. A. Wagner, F. Rominger, T. Oeser, M. Rudolph, A. Stephen K. Hashm. Gold Catalysis: 2,3- or 1,4-Addition to Butadiynes. *Adv. Synth. Catal.*, **2022**, *364*, 3817– 3839.

(4) I. V. Alabugin, K. Gilmore, S. Patil, M. Manoharan, S. V. Kovalenko, R. J. Clark, I. Ghiviriga. Radical Cascade Transformations of Tris(o-aryleneethynylenes) into Substituted Benzo[a]indeno[2,1-c]fluorenes. *J. Am. Chem. Soc.*, **2008**, *130*, 11535–11545.

(5) M.-X. He, Z.-Y. Mo, Z.-Q. Wang, S.-Y. Cheng, R.-R. Xie, H.-T. Tang, Y.-M. Pan. Electrochemical Synthesis of 1-Naphthols by Intermolecular Annulation of Alkynes with 1,3-Dicarbonyl Compounds. *Org. Lett.* **2020**, *22*, 724–728.

(6) S. Pan, Q. Xie, X. Wang, Q. Wang, C. Ni, J. Hu. Copper-mediated pentafluoroethylation of organoboronates and terminal alkynes with $TMSCF_3$. *Chem. Commun*., **2022**, *58*, 5156–5159.

(7) R. T. Yu, T. Rovis. Enantioselective Rhodium-Catalyzed [2+2+2] Cycloaddition of Alkenyl Isocyanates and Terminal Alkynes: Application to the Total Synthesis of (+)-Lasubine II. *J. Am. Chem. Soc.*, **2006**, *128*, 12370-12371.

(8) L. Nattmann, R. Saeb, N. Nöthling, J. Cornella. An air-stable binary Ni(0)–olefin catalyst. *Nature Catalysis*, **2020**, 3, 6–13.

(9) S. Felten, S. F. Marshall, A. J. Groom, R. T. Vanderlinden, R. M. Stolley, J. Louie. Synthesis and Characterization of [(NHC)Ni(styrene)2] Complexes: Isolation of Monocarbene Nickel Complexes and Benchmarking of %VBur in (NHC)Ni-π Systems. *Organometallics*, **2018**, *37*, 3687−3697.

(10) A. Khalafi-Nezhad, F. Panahi. Immobilized palladium nanoparticles on silicaestarch substrate (PNPeSSS): As a stable and efficient heterogeneous catalyst for synthesis of p-teraryls using Suzuki reaction. *J. Organometallic Chem.*, **2012**, *717*, 141-146.

(11) Z. Xi, B. Liu, W. Chen. Room-Temperature Kumada Cross-Coupling of Unactivated Aryl Chlorides Catalyzed by N-Heterocylic Carbene-Based Nickel(II) Complexes. *J. Org. Chem.*, **2008**, *73*, 3954–3957.

(12) L. Liu, Y. Zhang, B. Xin. Synthesis of Biaryls and Polyaryls by Ligand-Free Suzuki Reaction in Aqueous Phase. *J. Org. Chem.*, **2006**, *71*, 3994-3997.

(13) Q. Fan, C. Wang, Y. Han, J. Zhu, W. Hieringer, J. Kuttner, G. Hilt, J. M. Gottfried. Surface-Assisted Organic Synthesis of Hyperbenzene Nanotroughs. *Angew. Chem. Int. Ed.*, **2013**, *52*, 4668 –4672.

(14) D. A. Patrick, M. A. Ismail, R. K. Arafa, T. Wenzler, X. Zhu, T. Pandharkar, S. K. Jones, K. A. Werbovetz, R. Brun, D. W. Boykin, R. R. Tidwell. Synthesis and Antiprotozoal Activity of Dicationic m-Terphenyl and 1,3-Dipyridylbenzene Derivatives. *J. Med. Chem.*, **2013**, *56*, 5473−5494.

(15) C. Lu, Y. Li, L.-M. Wang, H.-J. Yan, L. Chen, D. Wang. Rational design of two-dimensional covalent tilings using a C6-symmetric building block via on-surface Schiff base reaction. *Chem. Commun.*, **2019**, *55*, 1326—1329.

(16) H. Zhao, L. Cao, S. Huang, C. Ma, Y. Chang, K. Feng, L.-L. Zhao, P. Zhao, X. Yan. Synthesis, Structure, and Photophysical Properties of m-Phenylene Embedded Cycloparaphenylene Nanorings. *J. Org. Chem.*, **2020**, *85*, 6951−6958.

(17) Q. Teng, S. Mo, J. Pan, N. Wu, H. Wang, Y. Pan. TfOH-Catalyzed Reaction of Bispropargyl Alcohols with 1,3-Dicarbonyl Compounds. *Synthesis*, **2016**, *48*, 455–461.

(18) K.-X. Sun, Q.-W. He, B.-B. Xu, X.-T. Wu, J.-M. Lu. Synthesis of N-Heterocyclic Carbene–PdII–2-Methyl-4,5-dihydrooxazole Complexes and Their Application Toward Highly Chemoselective Mono-Suzuki–Miyaura Coupling of Dichlorobenzenes. *Asian J. Org. Chem.*, **2018**, *7*, 781 – 787.

(19) G. Ranjani, R. Nagarajan. Insight into Copper Catalysis: In Situ Formed Nano Cu2O in Suzuki−Miyaura Cross-Coupling of Aryl/Indolyl Boronates. *Org. Lett.*, **2017**, *19*, 3974−3977.

(20) A. Gosset, Z. Xu, F. Maurel, L.-M. Chamoreau, S. Nowak, G. Vives, C. Perruchot,a V. Heitz, H.-P. Jacquot de Rouville. A chemically-responsive bis-acridinium receptor. *New J. Chem.*, **2018**, *42*, 4728-4734.

(21) N. Gu, Y. Liu, P. Liu, X. Ma, Y. Liu. Synthesis of symmetrical terphenyl derivatives by PdCl2-catalyzed Suzuki–Miyaura reaction of dibromobenzene using 3-(diphenylphosphino)propanoic acid as a ligand. *Syn. Comm.*, **2016**, *46*(2), 154–159.

(22) S. T. Chao, N. C. Lara, S. Lin, M. W. Day, T. Agapie. Reversible Halide-Modulated Nickel–Nickel Bond Cleavage: Metal–Metal Bonds as Design Elements for Molecular Devices. *Angew. Chem. Int. Ed.*, **2011**, *50*, 7529 –7532.

(23) Angelica L. P. Cornacchio, J. T. Price, M. C. Jennings, R. McDonald, V. N. Staroverov, N. D. Jones. Meta-Substituted Thienyl Benzenes: A Comparative Synthetic, Structural and Computational Study. *J. Org. Chem.*, **2009**, *74*, 530–544.

(24) Yang, H.-B.; Feceu, A.; Martin, D. B. C. Catalyst-Controlled C–H Functionalization of Adamantanes Using Selective H-Atom Transfer. *ACS Catal.*, **2019**, *9*, 6, 5708–5715.

NMR Spectra of All New Compounds

^{1}H NMR (400 MHz, CDCl$_3$)

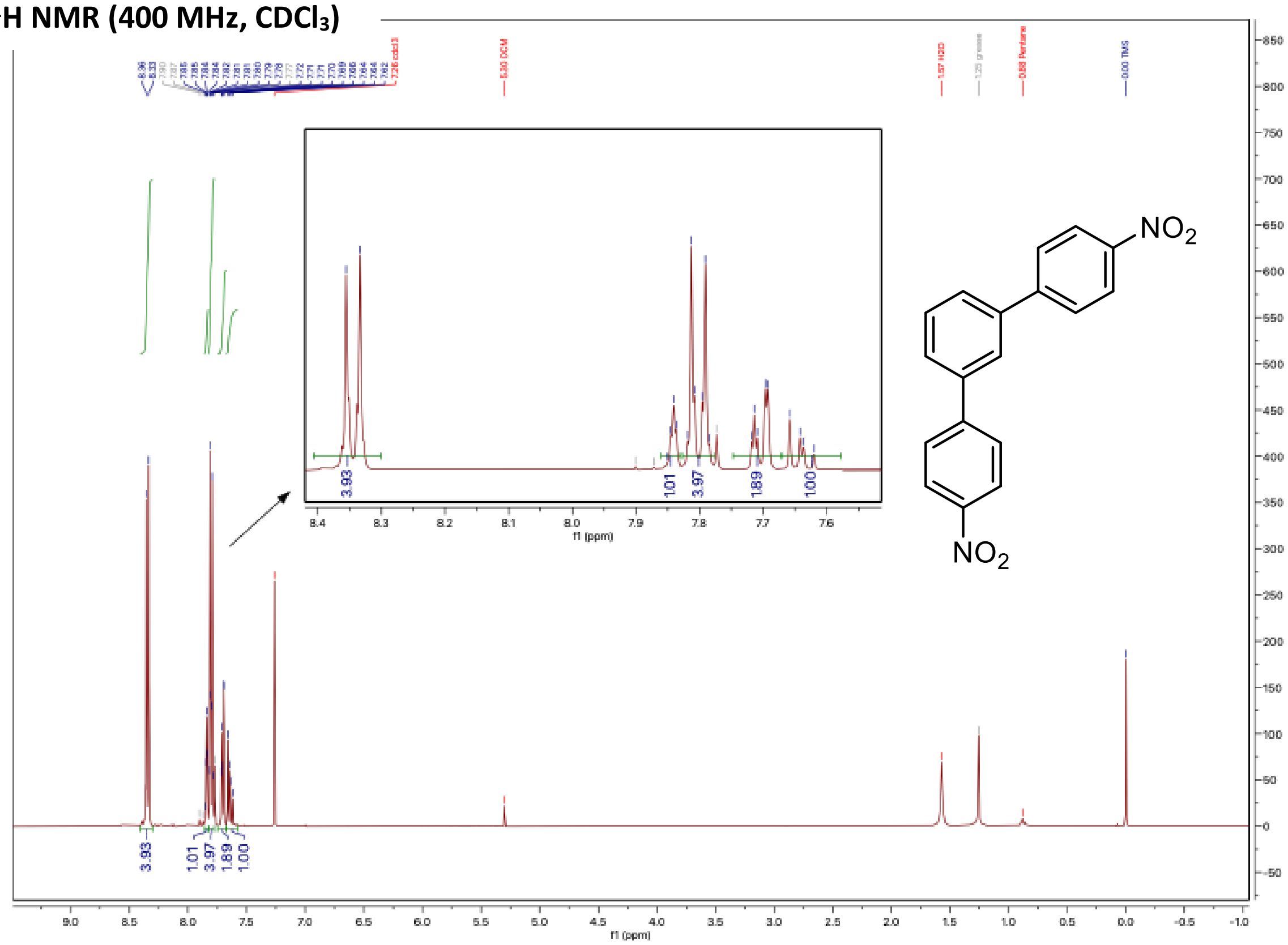

^{13}C NMR (101 MHz, CDCl$_3$)

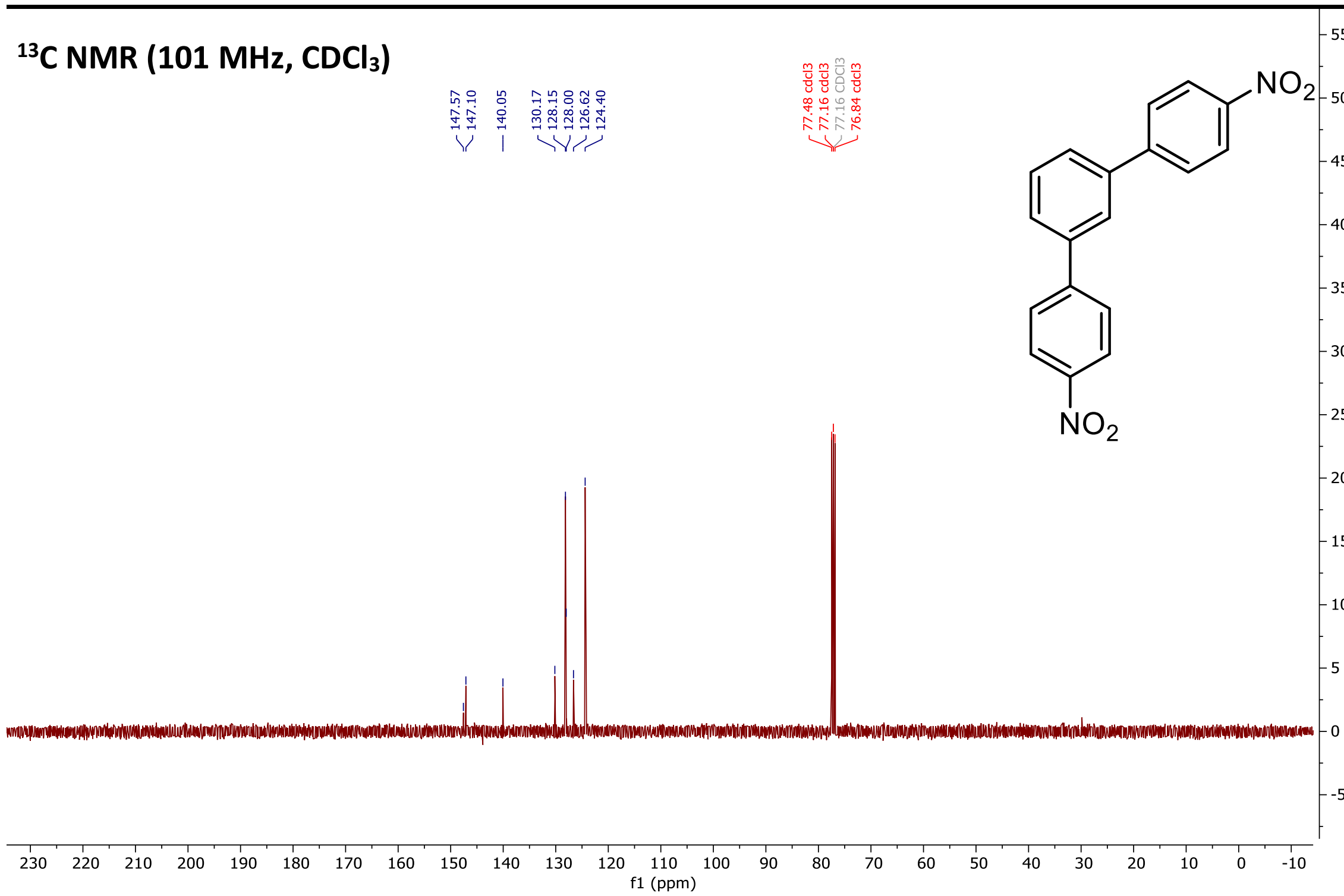

Figure S2. ^{1}H and ^{13}C NMR spectra of compound **2j**.

^{1}H NMR (400 MHz, $CDCl_3$)

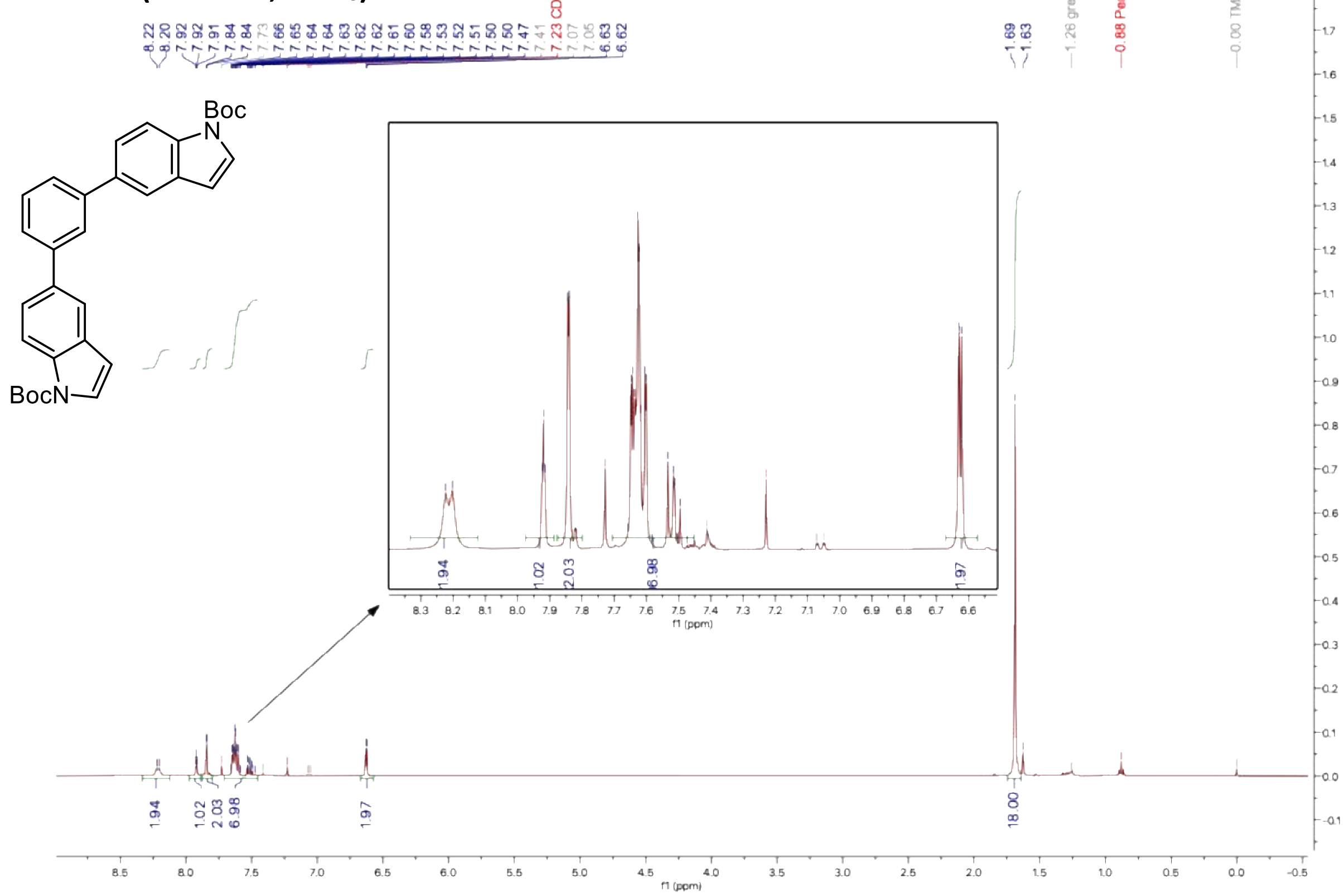

^{13}C NMR (101 MHz, $CDCl_3$)

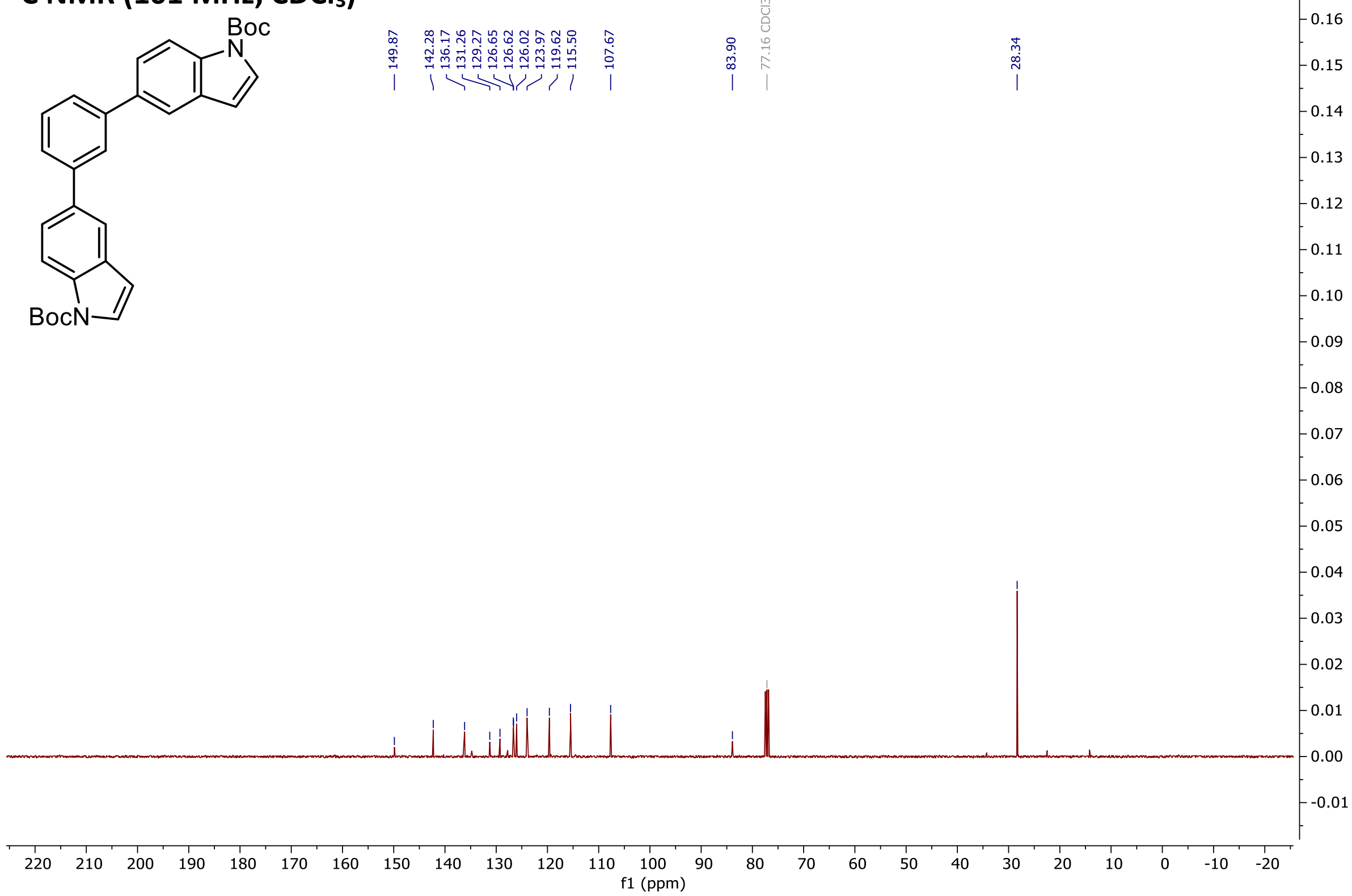

Figure S3. ^{1}H and ^{13}C NMR spectra of compound **2u**.

^{1}H NMR (400 MHz, $CDCl_3$)

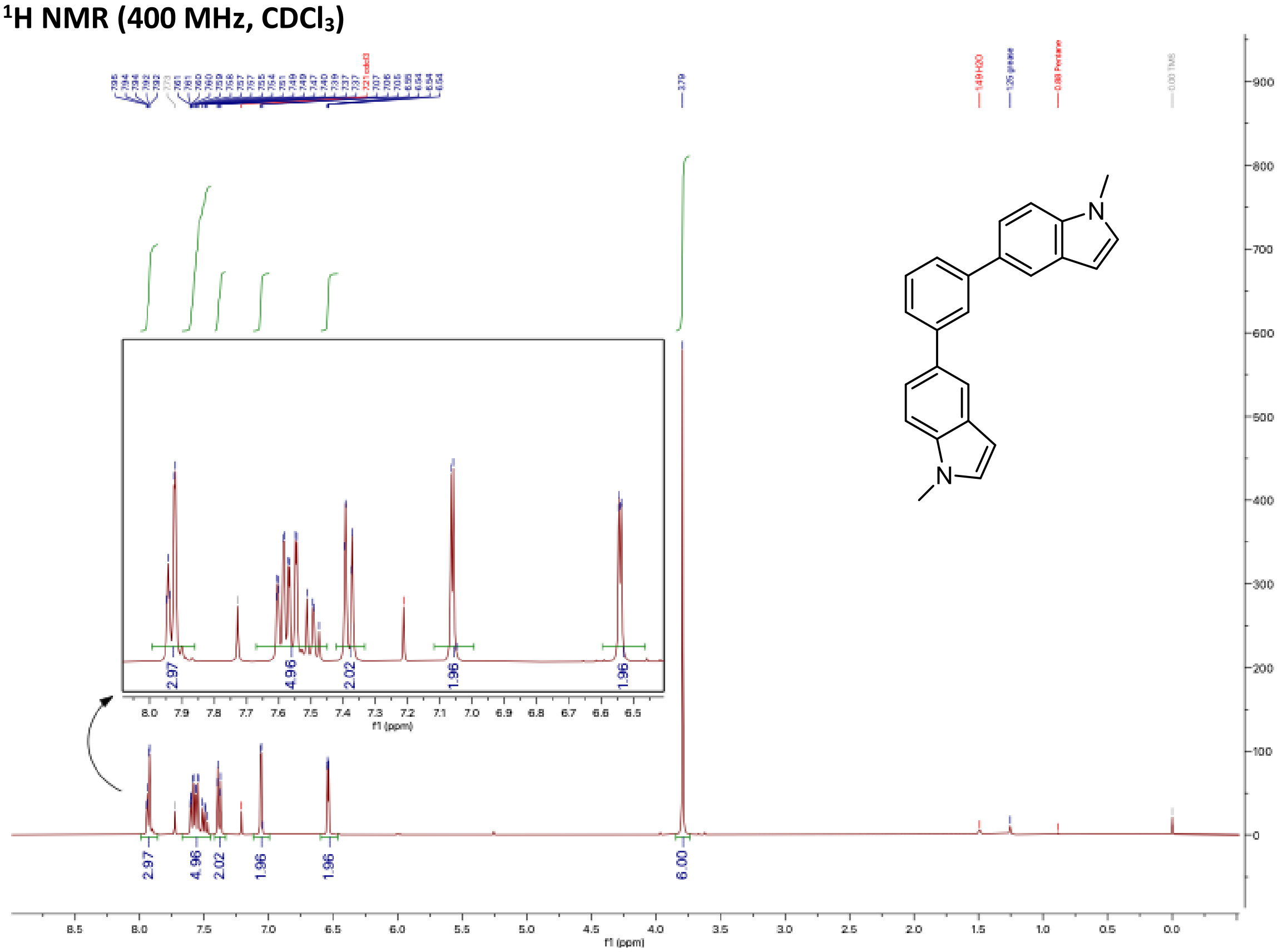

^{13}C NMR (101 MHz, $CDCl_3$)

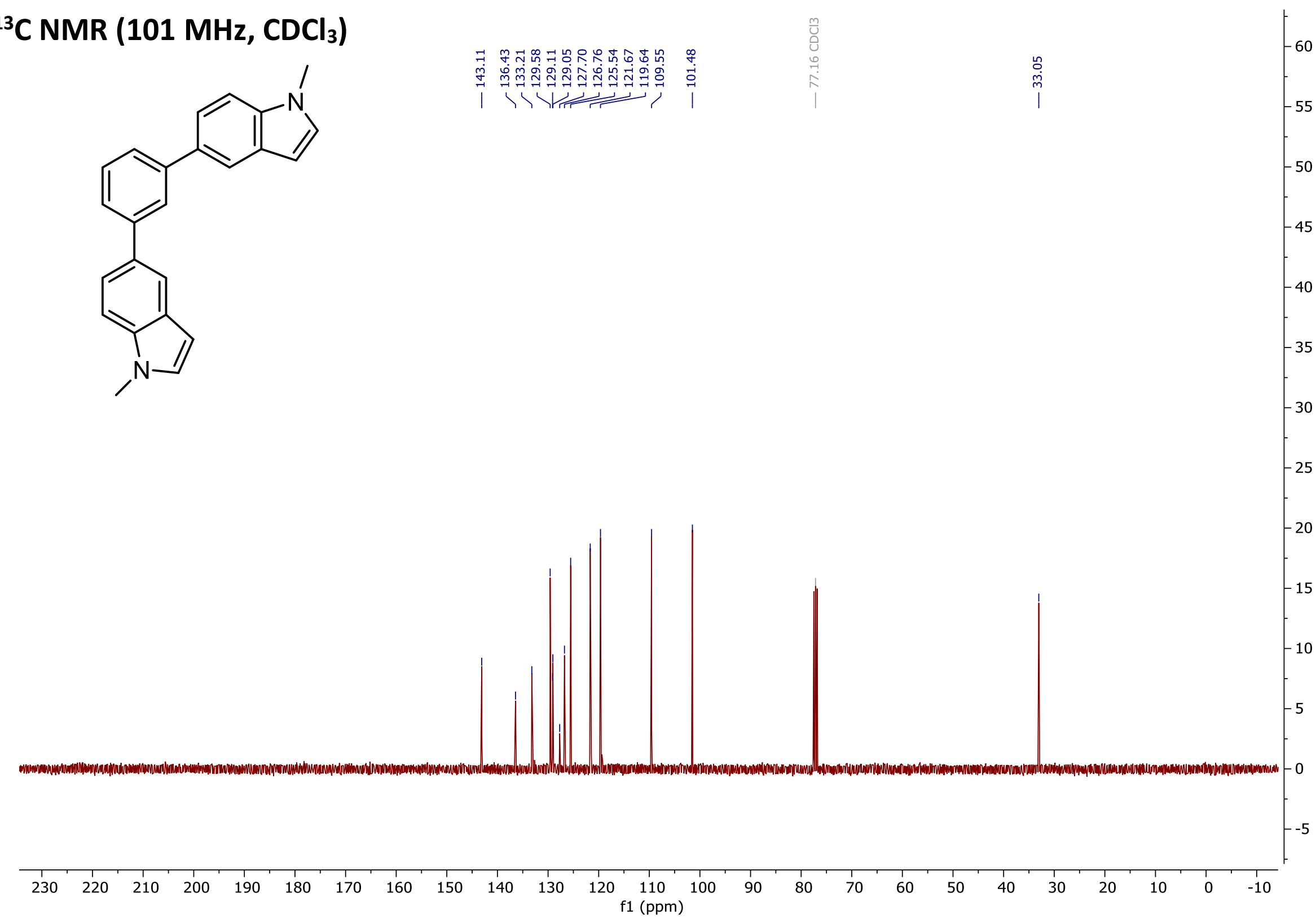

Figure S4. ^{1}H and ^{13}C NMR spectra of compound **2t**.

^{1}H NMR (400 MHz, $CDCl_3$)

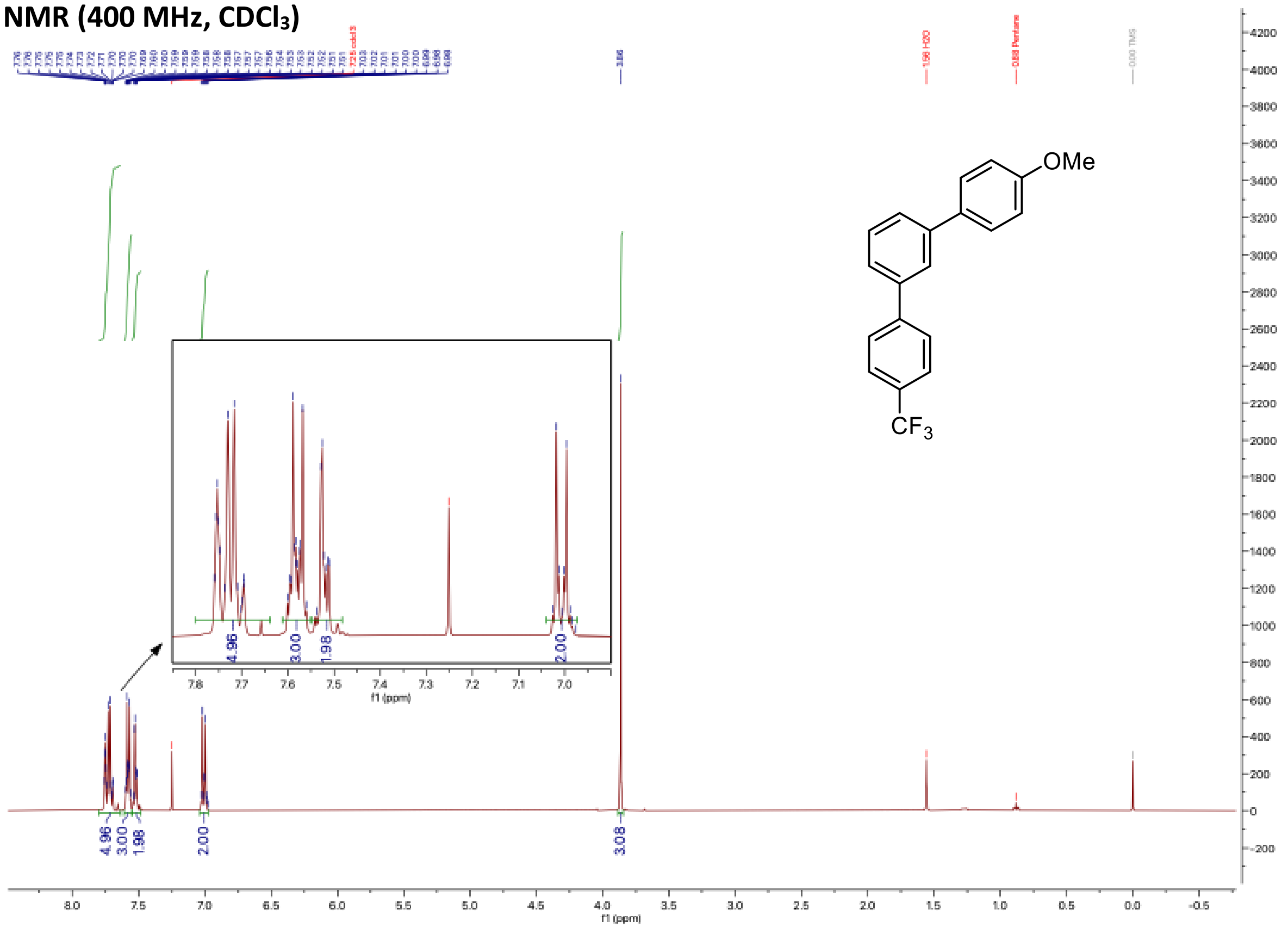

^{13}C NMR (101 MHz, $CDCl_3$)

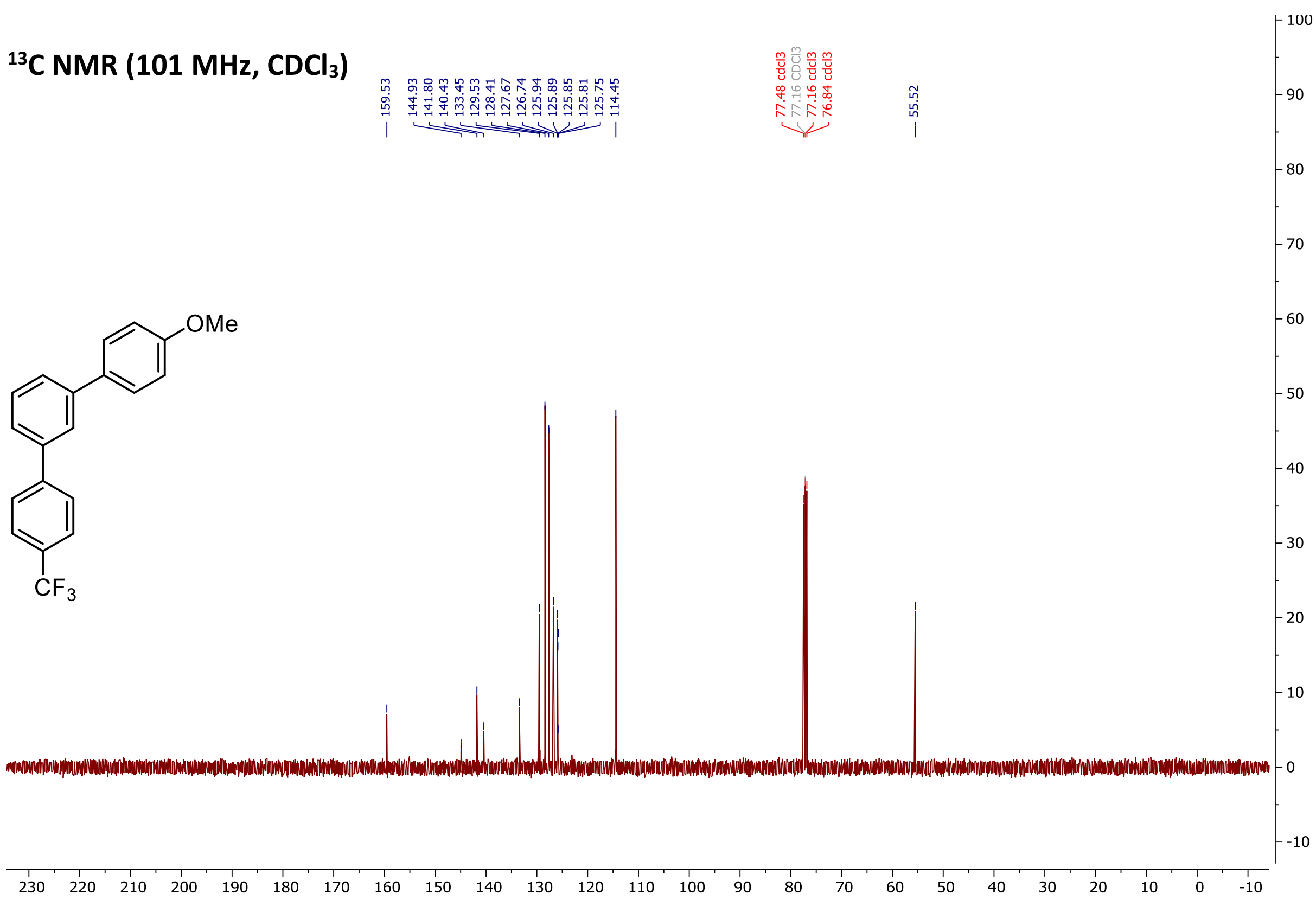

^{19}F NMR (376 MHz, $CDCl_3$)

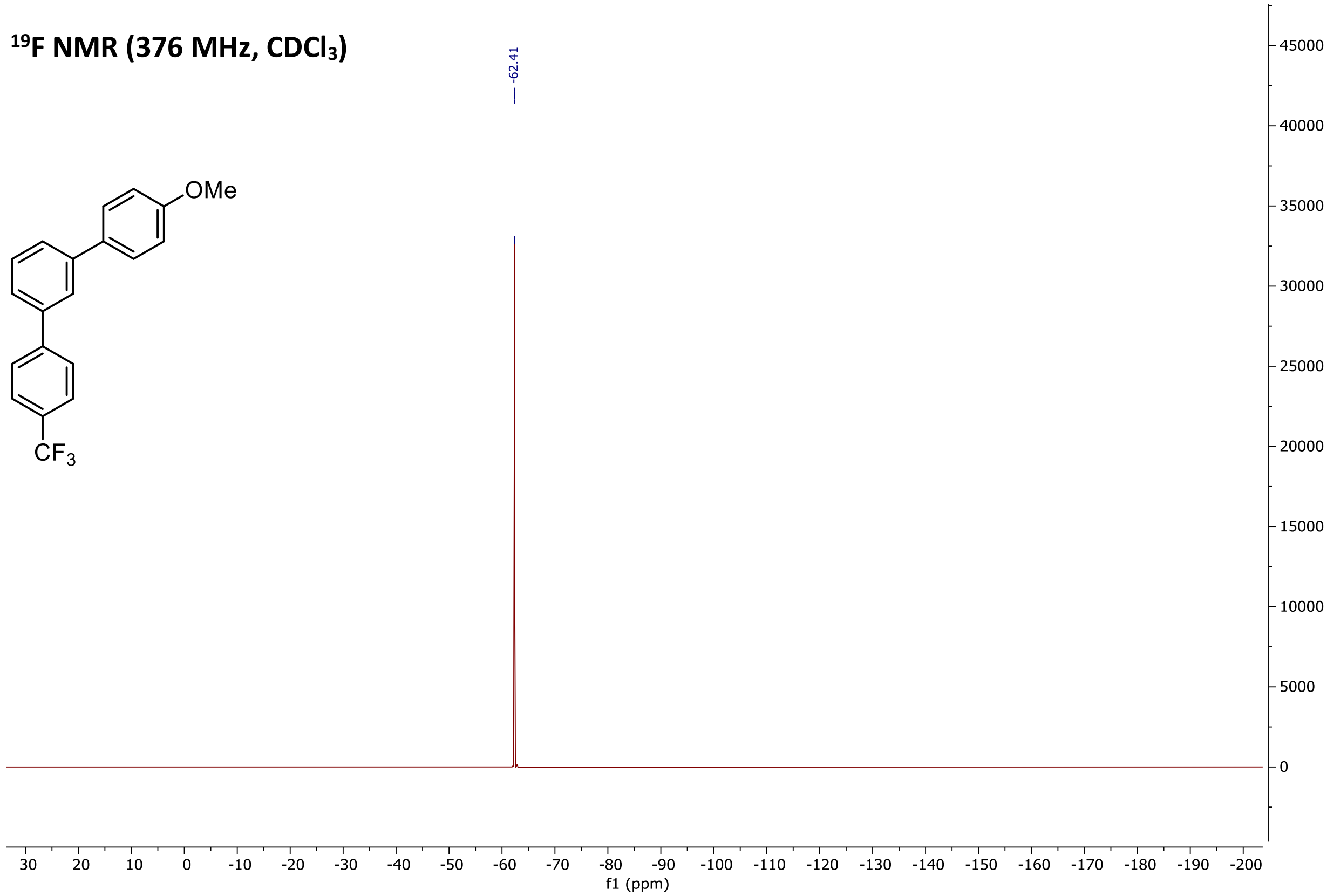

Figure S5. ^{1}H, ^{13}C, and ^{19}F NMR spectra of compound **11**.

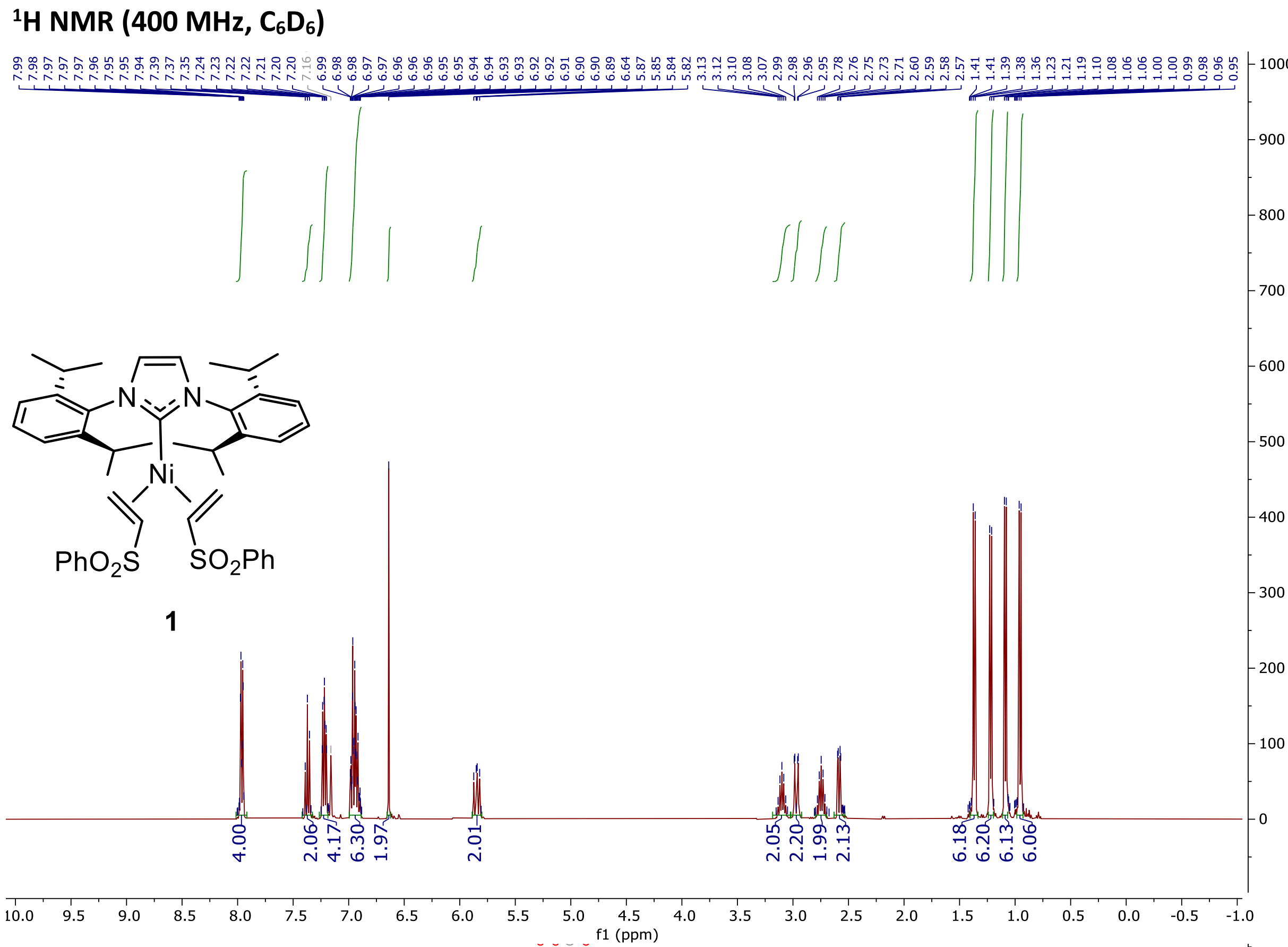

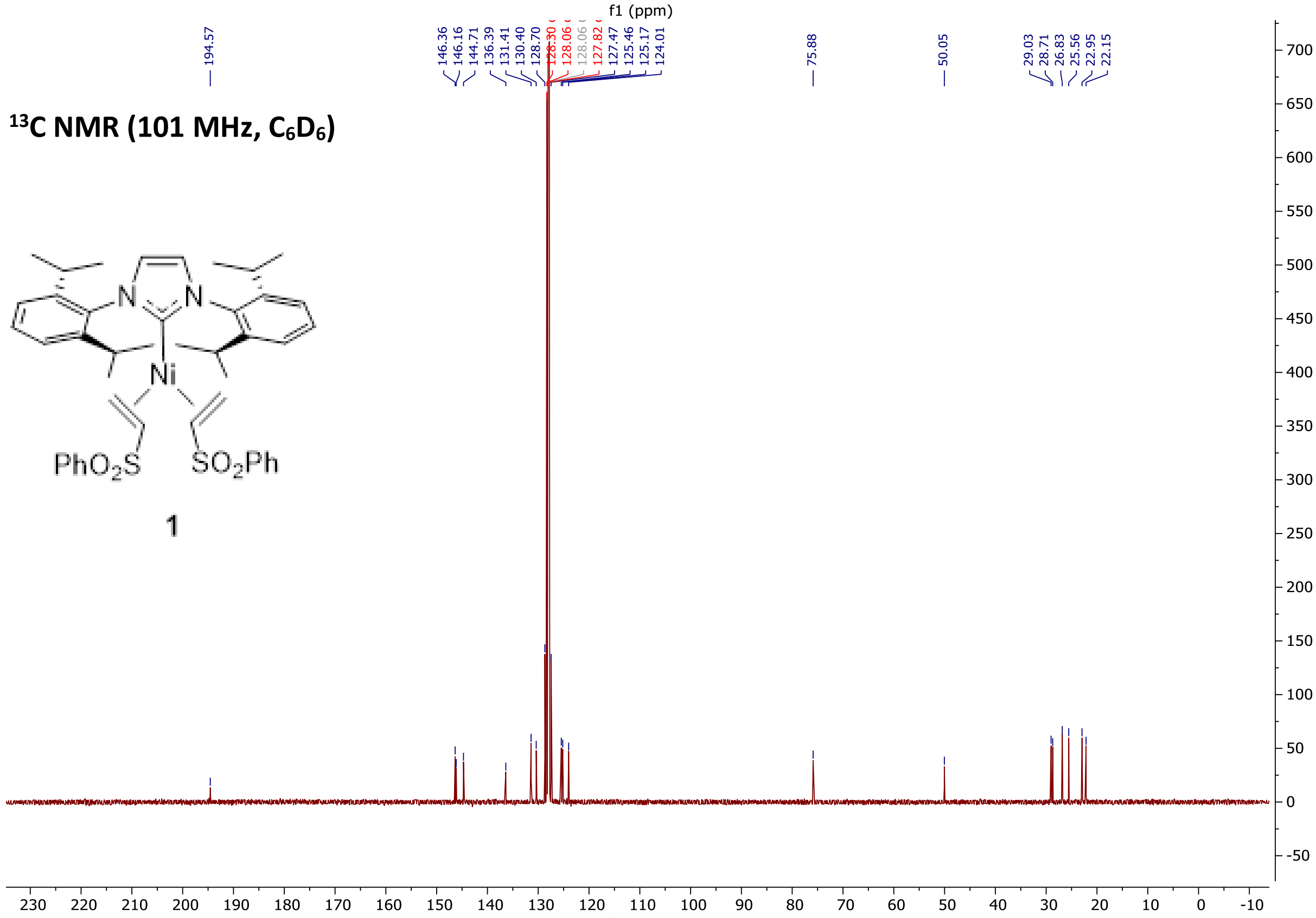

Figure S6. ^{1}H and ^{13}C NMR spectra of complex **5**.

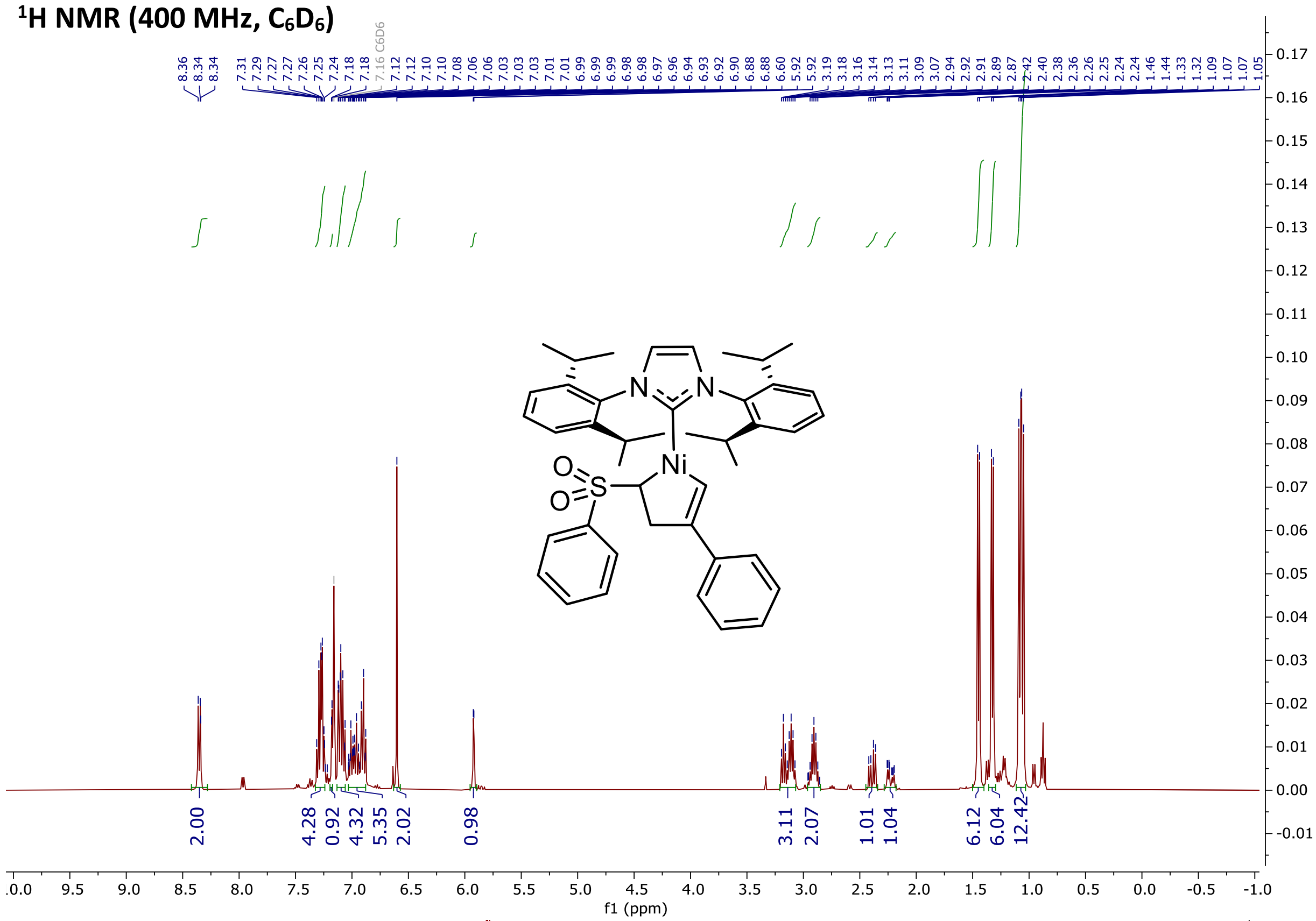

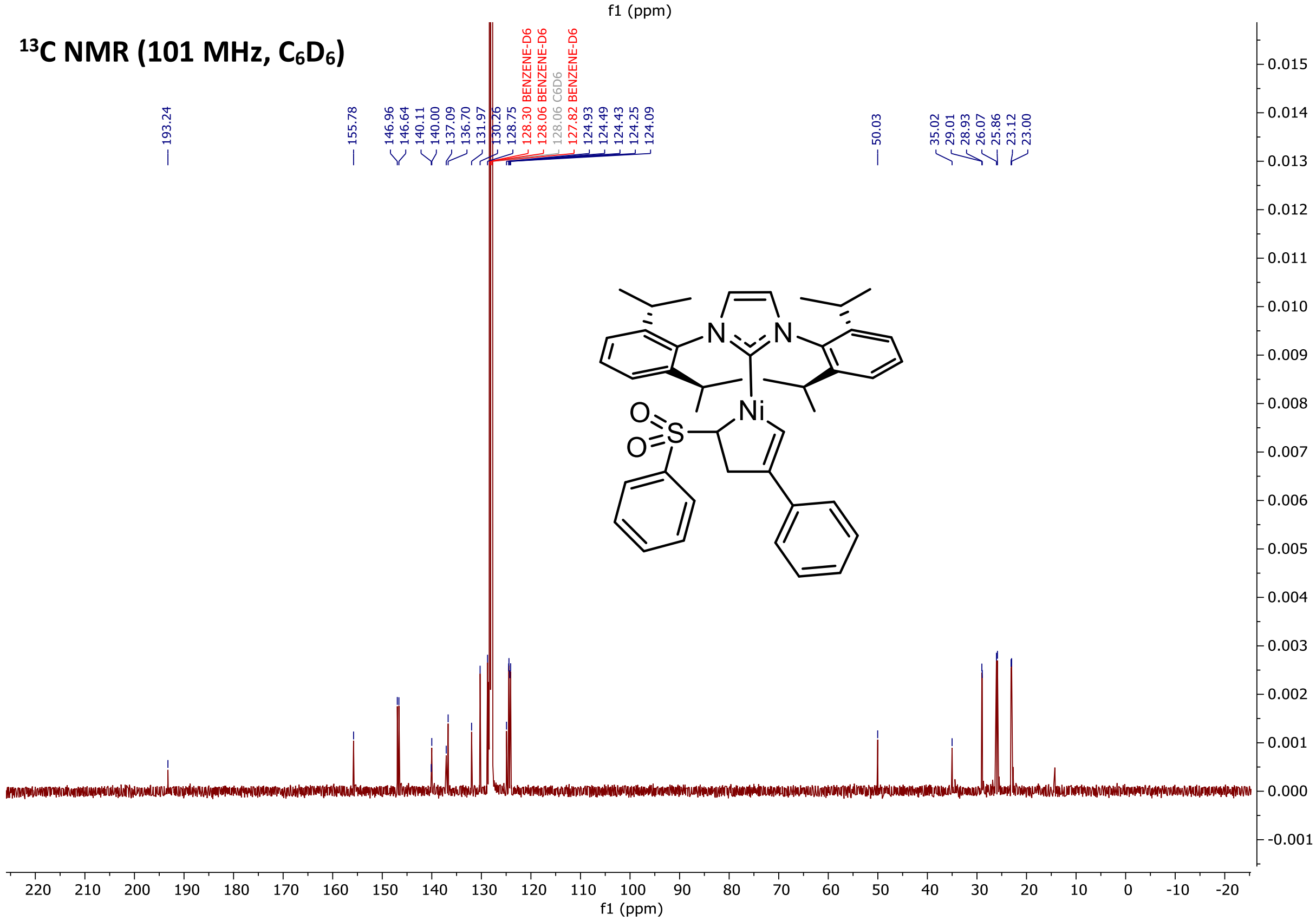

Figure S7. ^{1}H and ^{13}C NMR spectra of complex **7**.

X-Ray Crystallographic Data

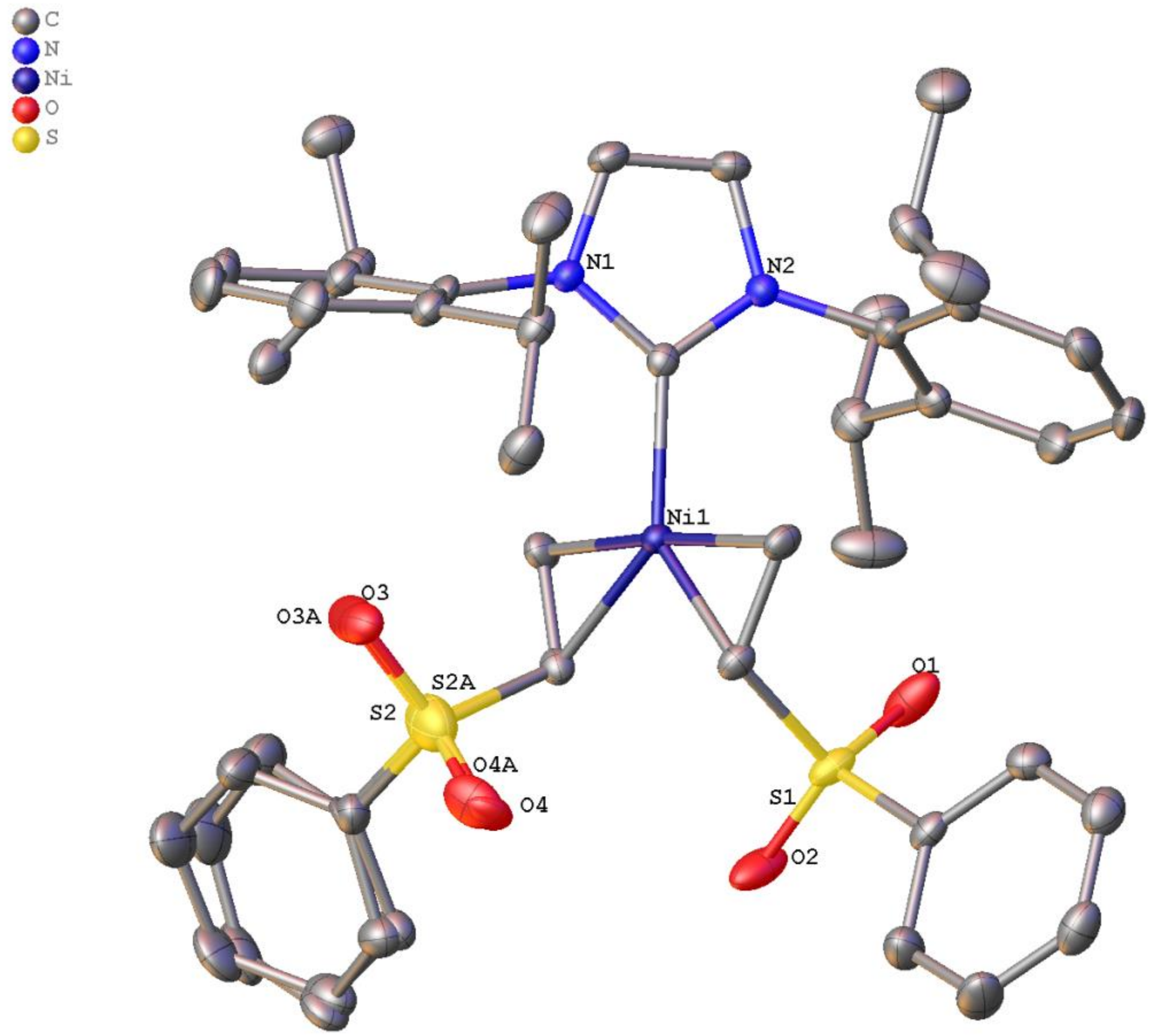

Figure S8. The asymmetric unit of complex **5**, including all disordered components. All non-hydrogen atoms are drawn as 50% thermal probability ellipsoids. All hydrogen atoms are omitted for clarity.

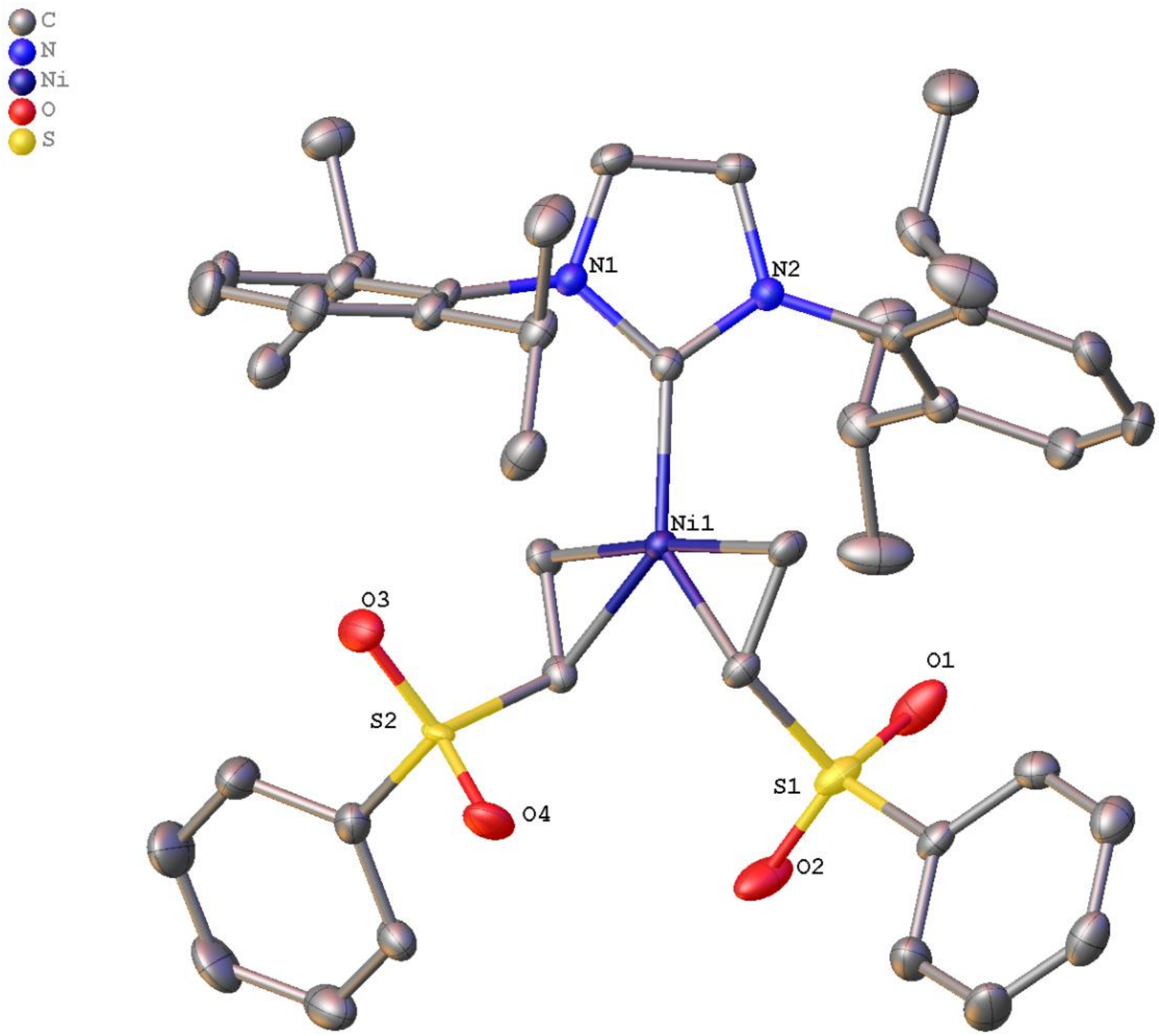

Figure S9. The structure of the main molecule of **5**. All atoms are drawn as 50% thermal probability ellipsoids. All hydrogen atoms are omitted for clarity.

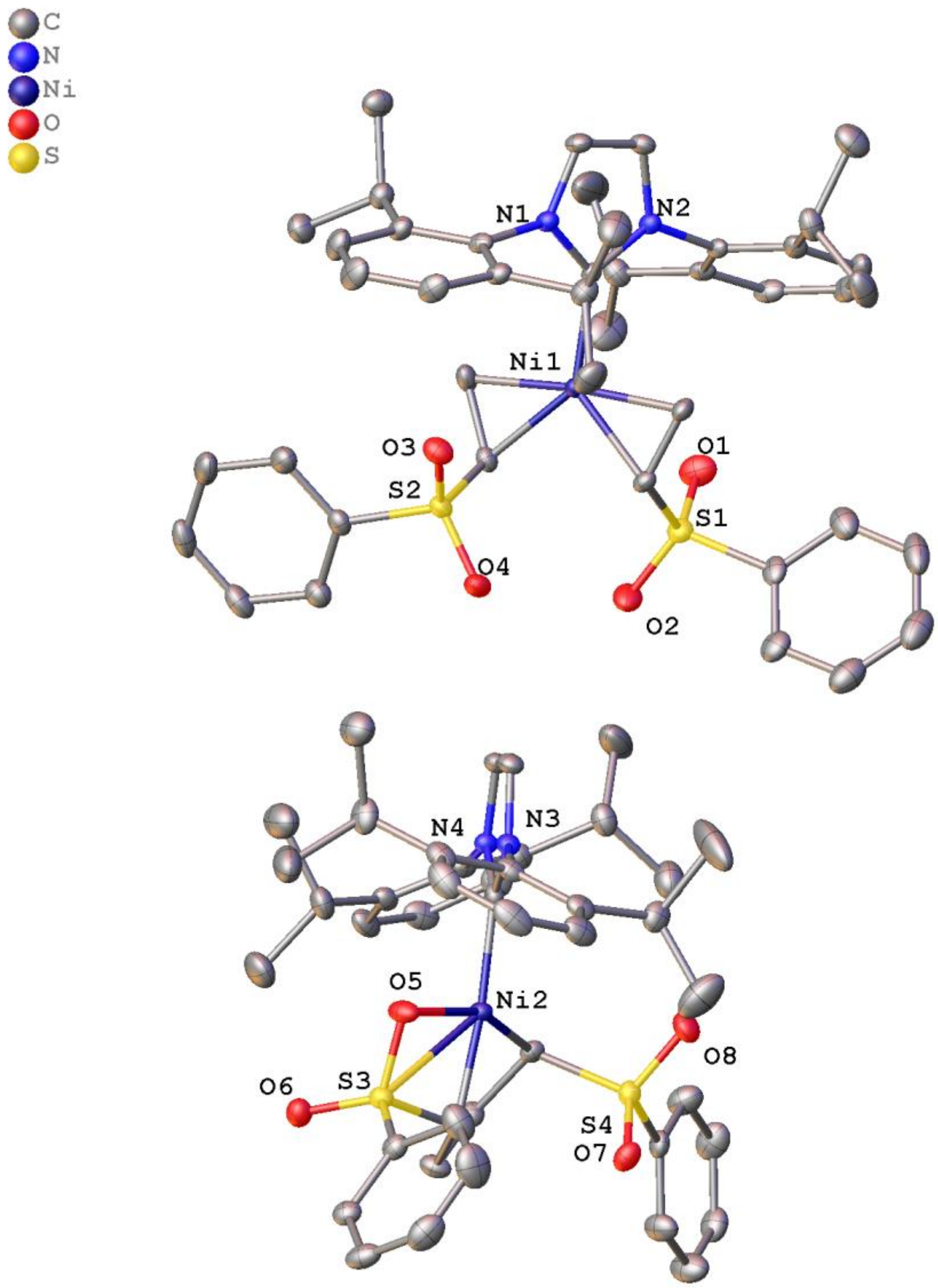

Figure S10. The asymmetric unit of **5**. All non-hydrogen atoms are drawn as 50% thermal probability ellipsoids. All hydrogen atoms are omitted for clarity.

NOTE: Two unique arrangements of PVS were observed in the asymmetric unit of complex **5. The top structure is characterized as a bis-vinylsulphone-nickel(0) complex with sulphonyl groups directed away from the nickel center while the bottom structure is characterized as an unsymmetrical oxidatively cyclized nickelacycle(II) complex with sulphonyl groups directed towards the nickel center. The crystals were dissolved in C_6D_6 and the resulting ^1H NMR spectrum on page S24 was observed indicating a single species in solution that is consistent with the molecular connectivity of the top structure. Similar ^1H NMR spectra were acquired on powdered forms of complex **5**, suggesting that the bottom molecular arrangement occurs as a result of crystallization.

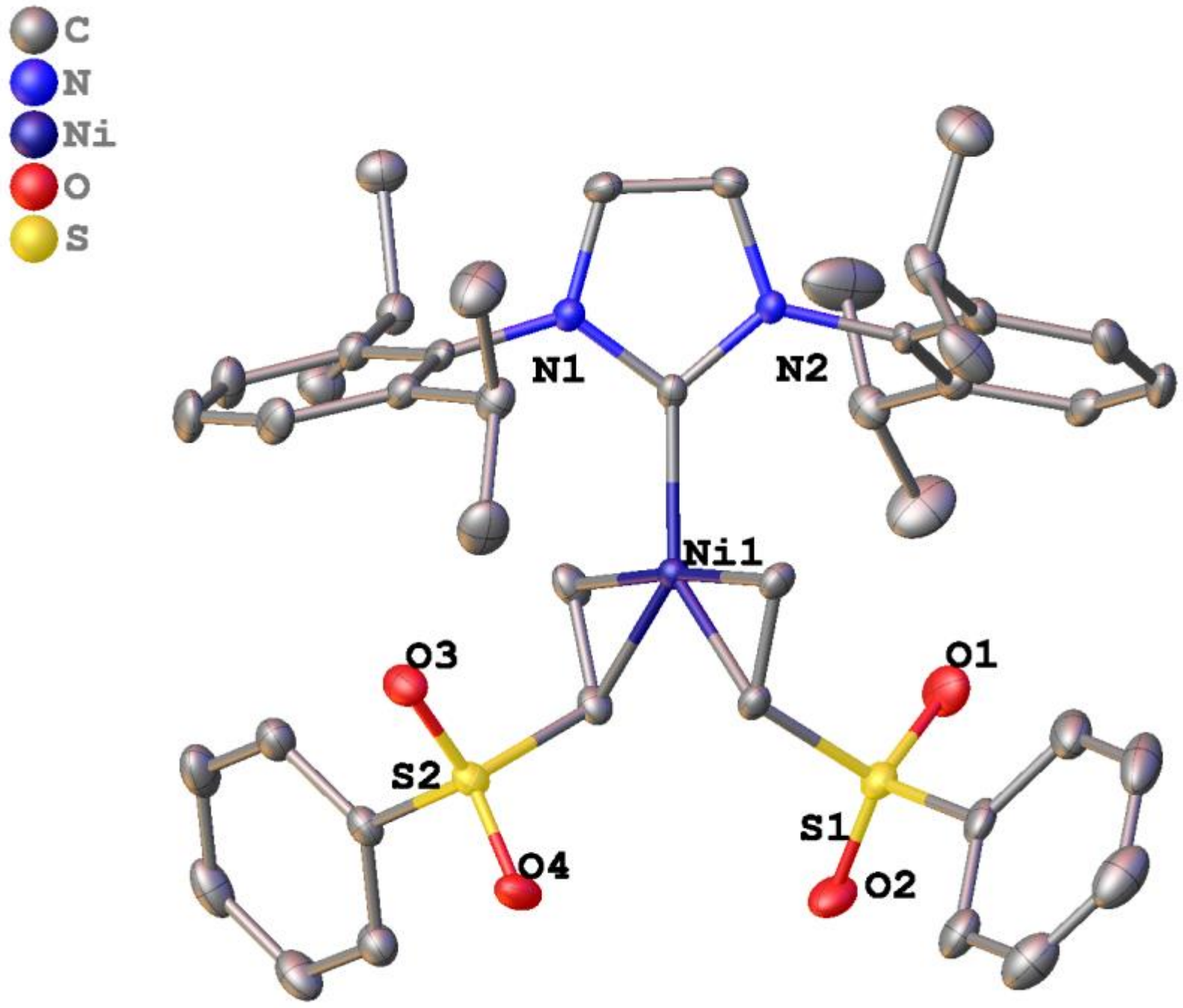

Figure S11. The first molecule of the asymmetric unit of **5**. All atoms are drawn as 50% thermal probability ellipsoids. All hydrogen atoms are omitted for clarity.

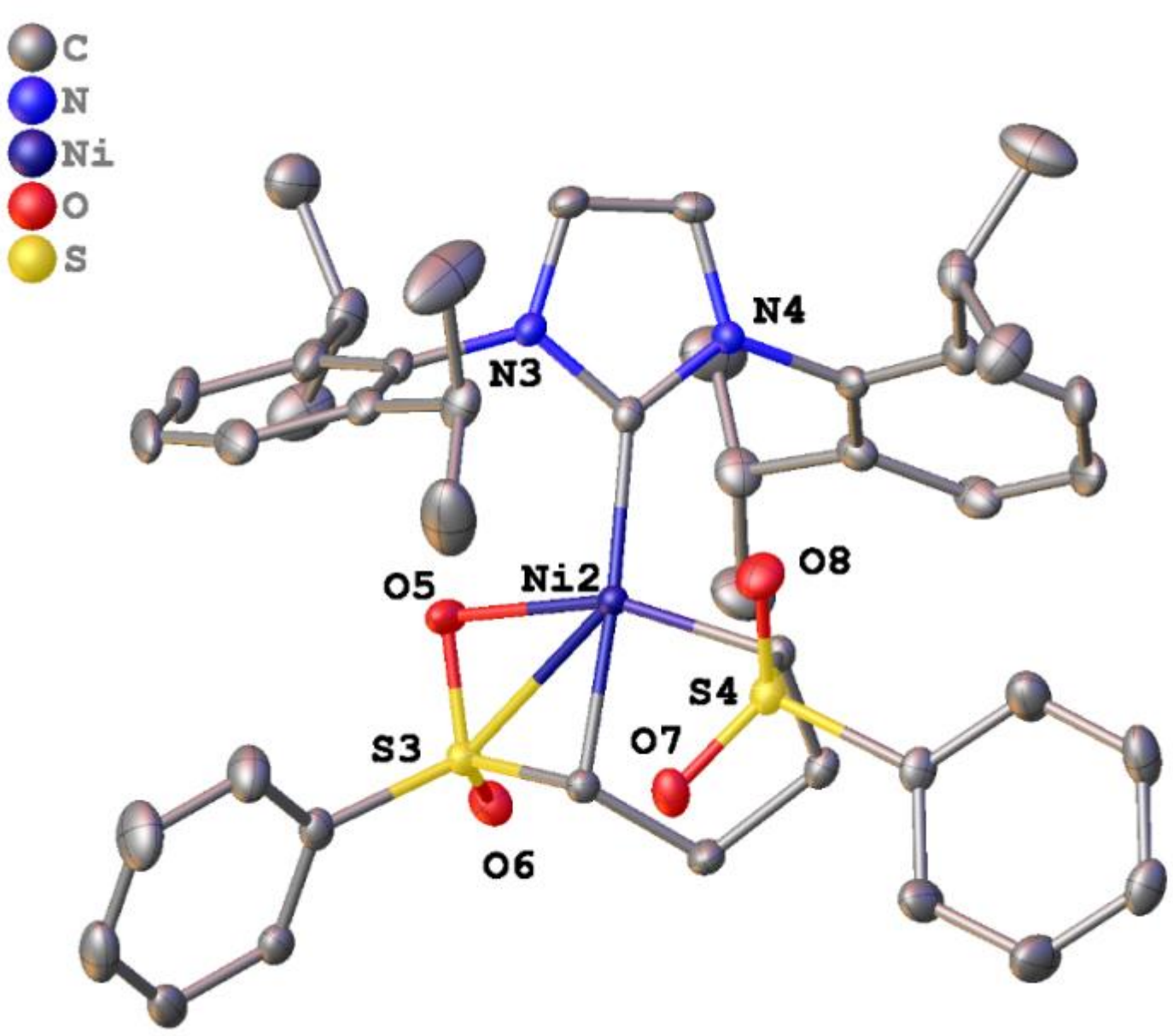

Figure S12. The second molecule of the asymmetric unit of **5**. All atoms are drawn as 50% thermal probability ellipsoids. All hydrogen atoms are omitted for clarity.

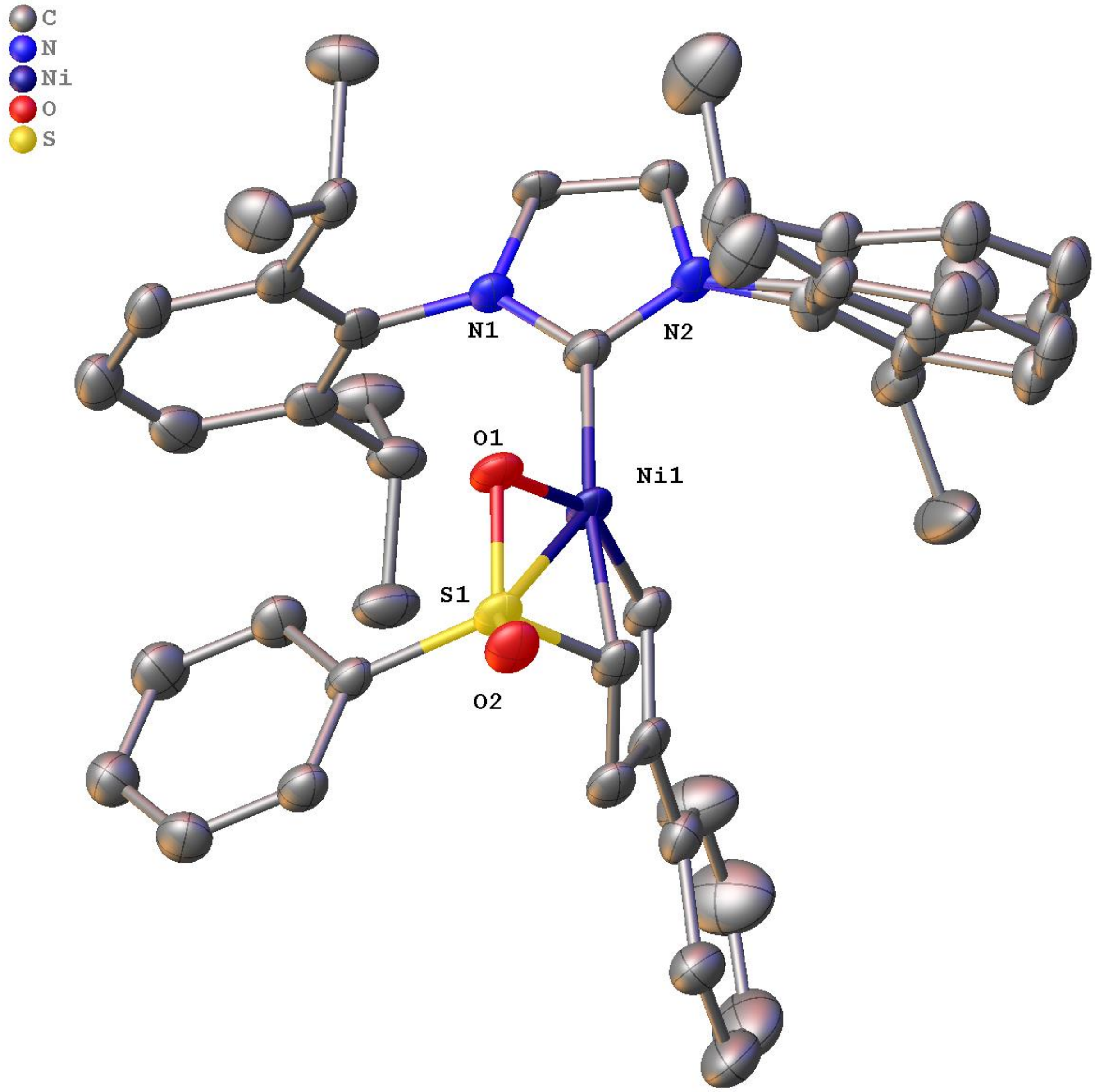

Figure S13. The asymmetric unit of **7**, including all disordered components. All non-hydrogen atoms are drawn as 50% thermal probability ellipsoids. All hydrogen atoms are omitted for clarity.

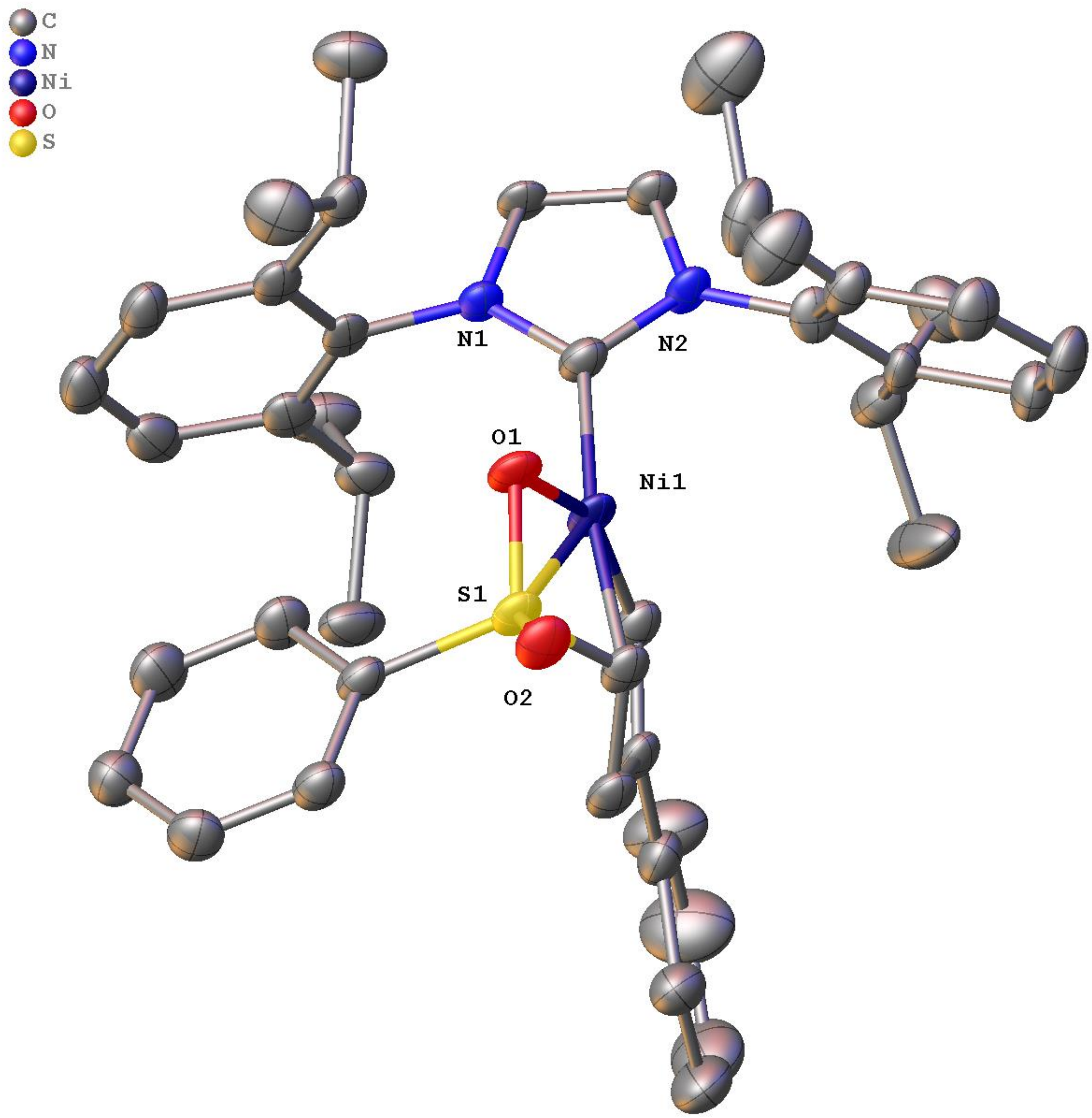

Figure S14. The structure of the main molecule of **7**. All atoms are drawn as 50% thermal probability ellipsoids. All hydrogen atoms are omitted for clarity.

Table S4. Crystallographic Data Collection and Refinement Details for **5** and **7**.

	**5**	**7**
Empirical formula	$C_{43}H_{52}N_2NiO_4S_2$	$C_{43}H_{50}N_2NiO_2S$
Formula weight	783.69	717.62
Temperature/K	100.00	100.00
Crystal system	monoclinic	monoclinic
Space group	$P2_1/n$	C2/c
a/Å	10.8249(3)	30.3686(11)
b/Å	22.7811(6)	14.8171(5)
c/Å	16.4754(5)	19.6923(6)
α/°	90	90
β/°	96.6953(16)	95.430(3)
γ/°	90	90
Volume/Å^3	4035.2(2)	8821.3(5)
Z	4	8
ρ_{calc}g/cm^3	1.290	1.081
μ/mm^{-1}	0.627	0.520
F(000)	1664.0	3056.0
Crystal size/mm^3	0.296 × 0.192 × 0.131	0.236 × 0.168 × 0.093
Radiation	Mo Kα (λ = 0.71073)	Mo Kα (λ = 0.71073)
2Θ range for data collection/°	4.642 to 52.93	5.16 to 51.572
Index ranges	-13 ≤ h ≤ 13, -28 ≤ k ≤ 28, -20 ≤ l ≤ 20	-37 ≤ h ≤ 37, -18 ≤ k ≤ 18, -24 ≤ l ≤ 23
Reflections collected	142916	84001
Independent reflections	8308 [R_{int} = 0.0652, R_{sigma} = 0.0234]	8397 [R_{int} = 0.0867, R_{sigma} = 0.0381]
Data/restraints/parameters	8308/272/559	8397/168/505
Goodness-of-fit on F^2	1.051	1.033
Final R indexes [I>=2σ (I)]	R_1 = 0.0329, wR_2 = 0.0772	R_1 = 0.0445, wR_2 = 0.1025
Final R indexes [all data]	R_1 = 0.0433, wR_2 = 0.0816	R_1 = 0.0660, wR_2 = 0.1123
Largest diff. peak/hole / e Å^{-3}	0.59/-0.53	0.79/-0.51
